# Plant-growth promotion by proteobacterial strains depends on the availability of phosphorus and iron in *Arabidopsis thaliana* plants

**DOI:** 10.3389/fmicb.2022.1083270

**Published:** 2022-12-13

**Authors:** Daniela Orellana, Daniel Machuca, Miguel Angel Ibeas, José Manuel Estevez, María Josefina Poupin

**Affiliations:** ^1^Laboratorio de Bioingeniería, Facultad de Ingeniería y Ciencias, Universidad Adolfo Ibáñez, Santiago, Chile; ^2^Center of Applied Ecology and Sustainability (CAPES), Santiago, Chile; ^3^ANID - Millennium Science Initiative Program - Millennium Nucleus for the Development of Super Adaptable Plants (MN-SAP), Santiago, Chile; ^4^Centro de Biotecnología Vegetal, Facultad de Ciencias de la Vida, Universidad Andres Bello, Santiago, Chile; ^5^Fundación Instituto Leloir and IIBBA-CONICET, Buenos Aires, Argentina

**Keywords:** plant-growth promoting bacteria, phosphate, iron, PSR, beneficial bacteria, plant nutrition, plant microbiome, nutrient deficiency

## Abstract

Phosphorus (as phosphate, Pi) and iron (Fe) are critical nutrients in plants that are often poorly available in the soil and can be microbially affected. This work aimed to evaluate how plant-rhizobacteria interaction changes due to different Pi or Fe nutritional scenarios and to study the underlying molecular mechanisms of the microbial modulation of these nutrients in plants. Thus, three proteobacteria (*Paraburkholderia phytofirmans* PsJN, *Azospirillum brasilense* Sp7, and *Pseudomonas putida* KT2440) were used to inoculate Arabidopsis seeds. Additionally, the seeds were exposed to a nutritional factor with the following levels for each nutrient: sufficient (control) or low concentrations of a highly soluble source or sufficient concentrations of a low solubility source. Then, the effects of the combinatorial factors were assessed in plant growth, nutrition, and genetic regulation. Interestingly, some bacterial effects in plants depended on the nutrient source (e.g., increased aerial zones induced by the strains), and others (e.g., decreased primary roots induced by Sp7 or KT2440) occurred regardless of the nutritional treatment. In the short-term, PsJN had detrimental effects on plant growth in the presence of the low-solubility Fe compound, but this was not observed in later stages of plant development. A thorough regulation of the phosphorus content was detected in plants independent of the nutritional treatment. Nevertheless, inoculation with KT2440 increased P content by 29% Pi-deficiency exposed plants. Conversely, the inoculation tended to decrease the Fe content in plants, suggesting a competition for this nutrient in the rhizosphere. The P-source also affected the effects of the PsJN strain in a double mutant of the phosphate starvation response (PSR). Furthermore, depending on the nutrient source, PsJN and Sp7 strains differentially regulated PSR and IAA- associated genes, indicating a role of these pathways in the observed differential phenotypical responses. In the case of iron, PsJN and SP7 regulated iron uptake-related genes regardless of the iron source, which may explain the lower Fe content in inoculated plants. Overall, the plant responses to these proteobacteria were not only influenced by the nutrient concentrations but also by their availabilities, the elapsed time of the interaction, and the specific identities of the beneficial bacteria.

**Graphical Abstract:**
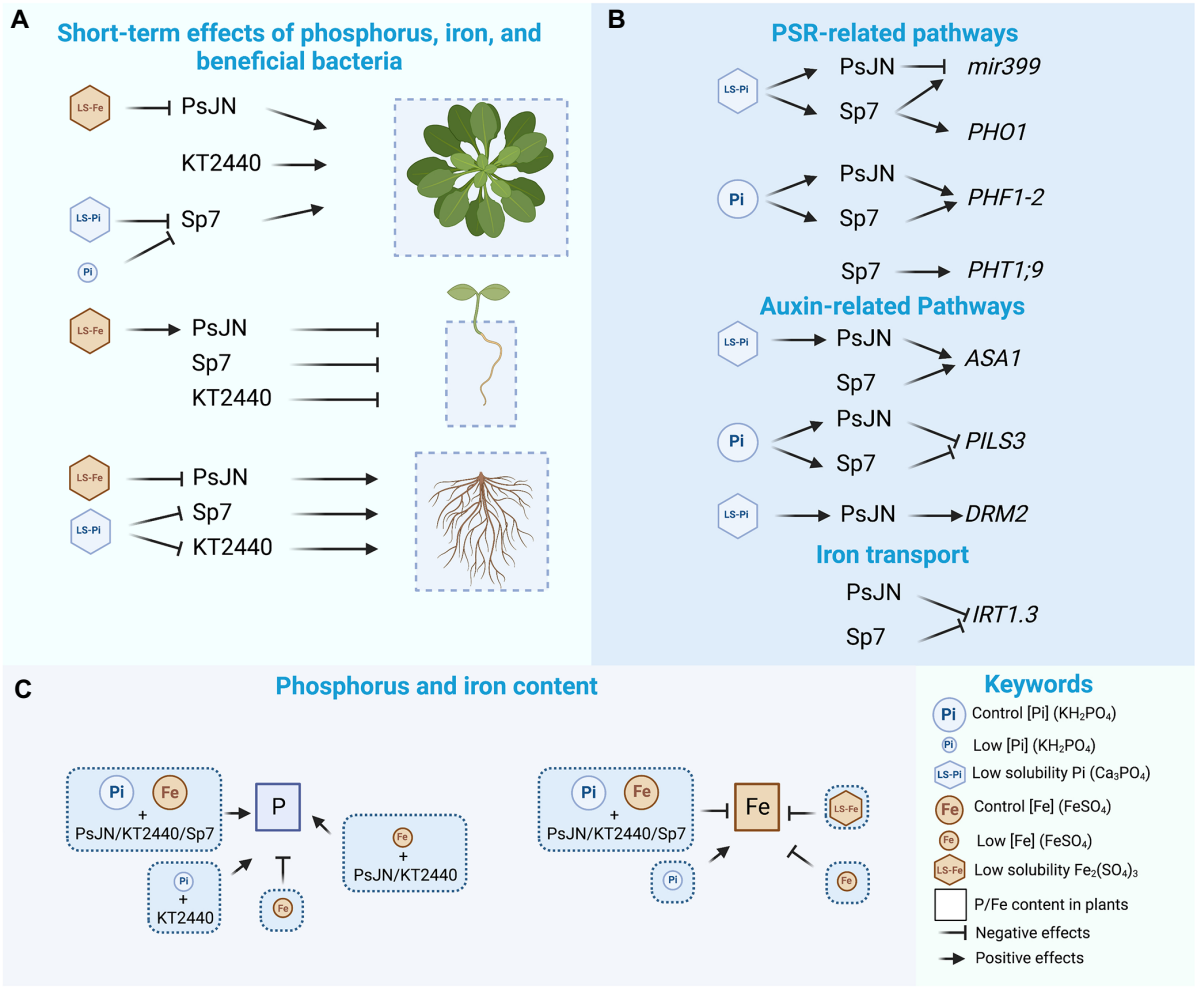
The effects of the different nutritional and inoculation treatments are indicated for plant growth parameters **(A)**, gene regulation **(B)** and phosphorus and iron content **(C)**. Figures created with BioRender.com with an academic license.

The effects of the different nutritional and inoculation treatments are indicated for plant growth parameters **(A)**, gene regulation **(B)** and phosphorus and iron content **(C)**. Figures created with BioRender.com with an academic license.

## Introduction

Nutrient deficiency in most terrestrial ecosystems constrains global primary productivity in both natural and agricultural environments, threatening food security for a growing population and the achievement of the Sustainable Development Goals (SDGs). An optimum supply of nutrients is essential for the normal growth, yield, and quality of crops (Ceasar et al., 2022). Although nutrients could be present in sufficient amounts in some soils, they usually have low bioavailability due to diverse biological and physical processes that impair their uptake by plants (Raghothama and Karthikeyan, 2005; Rengel and Marschner, 2005). Thus, it is important to understand how these processes operate and if they can be optimized, especially in a context of a changing climate.

Phosphorus (P) is an essential macronutrient for plants that plays a crucial role in photosynthesis, as a backbone of nucleic acids, in membranes as a component of phospholipids, and by participating in countless energy-dependent metabolic processes (Barragán-Rosillo et al., 2021; Poirier et al., 2022). Plants mainly acquire this element as orthophosphate (Pi) in its predominant form, H_2_PO_4_ (Ullrich-Eberius et al., 1984; Schachtman et al., 1998; Hammond et al., 2003). It is estimated that Pi deficiency is a major abiotic stress that limits crop productivity on 30%–40% of the world’s arable land (von Uexküll and Mutert, 1995; Vance et al., 2003; Wissuwa et al., 2005). In addition to areas of low absolute soil-P content, its deficiency is exacerbated because the element is strongly incorporated into insoluble soil particles, becoming less available to plants, or converted by microbial activity into organic forms not readily taken up by the plant (López-Arredondo et al., 2014; Barragán-Rosillo et al., 2021). Thus, P can be eroded and lost in run-off, causing eutrophication and pollution (Zak et al., 2018; Han et al., 2022). Nonmycorrhizal plants have sophisticated mechanisms to cope with Pi scarcity and to regulate its uptake and homeostasis in a group of responses known as the phosphate starvation response (PSR), which collectively increase their capacities to survive and reproduce in soils with low Pi availability (Raghothama, 1999; Abel et al., 2002). PSR includes changes in root system architecture (root/shoot ratio and proliferation of long root hairs); the upregulation of high-affinity Pi transporters; recycling of internal phosphate; root exudation, and the expression of genes encoding enzymes that facilitate the uptake of organic sources of Pi (Williamson et al., 2001; Wu et al., 2003; López-Arredondo et al., 2014; Crombez et al., 2019; Barragán-Rosillo et al., 2021). In Arabidopsis, Pi deficiency response is regulated by two transcription factors (TF) belonging to the MYB family, PHOSPHATE STARVATION RESPONSE1 (PHR1) and PHR1-like1 (PHL1). Those TF have an important role in phosphate sensing and regulate the transport and re-mobilization coding genes (Bustos et al., 2010).

Iron (Fe) is an essential micronutrient for plants and animals and participates in vital cellular functions requiring redox reactions such as photosynthesis and respiration. This is given to its ability to cycle between its two oxidation states: Fe^2+^ and Fe^3+^ (Riaz and Guerinot, 2021). Perturbations of Fe uptake, transport, or storage influence plant growth and crop yield (Connorton et al., 2017). Although Fe is one of the most abundant elements found on earth, its availability is limited in the soil by pH and oxygen (Guerinot and Yi, 1994). In soils, it is found predominantly in the form of Fe^3+^, mainly as insoluble ferric hydroxides, with extremely low solubility (Brumbarova et al., 2008; Colombo et al., 2014; Vélez-Bermúdez and Schmidt, 2022). Fe bioavailability is further reduced in alkaline soil, which comprises one-third of the world’s arable land (Guerinot and Yi, 1994; Riaz and Guerinot, 2021). Depending on its abundance, Fe quantities can shift from inadequate to toxic for plants. For instance, a Fe deficiency generates leaf chlorosis, arrested growth, and decreased fitness (Briat et al., 1995; Kim and Guerinot, 2007). Thus, plants have complex regulatory mechanisms to adjust the uptake and distribution of iron to maintain Fe homeostasis (Kobayashi and Nishizawa, 2012; Liang, 2022; Vélez-Bermúdez and Schmidt, 2022). As among other macro and micronutrients, crosstalk between Pi and Fe has been reported, indicating that Pi could negatively regulate the accumulation of Fe (Hirsch et al., 2006; Zheng et al., 2009; Bouain et al., 2019; Bechtaoui et al., 2021; Zheng and Liu, 2021).

Nutrients have special cycling and dynamics in the rhizosphere product of the plant-microbial interactions occurring there (Carvalhais et al., 2013; Kuzyakov and Blagodatskaya, 2015). Thus, the use of mineral solubilizing and mobilizing microorganisms as single strains or synthetic communities has been suggested as an environmentally friendly approach to improving the nutritional status of plants (Schütz et al., 2018; Oleńska et al., 2020; Marín et al., 2021; Chaudhary et al., 2022; Devi et al., 2022). Nevertheless, little is known about how plant-microbial interaction changes due to chemical compounds with different solubilities or bioavailability. In addition, the molecular mechanisms underlying the microbial modulation of Pi or Fe homeostasis in plants are scarcely understood. With the aim of tackling these issues, three rhizobacteria (*Paraburkholderia phytofirmans* PsJN, *Pseudomonas putida* KT2440, and *Azospirillum brasilense* Sp7) representing genera with different P and Fe solubilization capacities and plant growth-promoting traits were evaluated in plants. These strains were used to inoculate *Arabidopsis thaliana* as a model of nonmycorrhizal plants. Several Pi and Fe status scenarios were used, combining compounds with different solubilities and bioavailability. Plant growth, stress parameters, transcriptional responses, and *Arabidopsis phr1* and *phl1* mutant genotypes (related to the Pi starvation responses) were analyzed in order to evaluate plants growing under specific nutritional scenarios concomitantly with the inoculation with single strains. Thus, a possible interaction between the nutrient source/availability and the different strains was assessed to understand the bacterial modulation of Arabidopsis phenotypical and molecular responses to different P and Fe contexts.

## Materials and methods

### Plant growth conditions and nutritional treatments

The proteobacteria *Paraburkholderia phytofirmans* PsJN, *Azospirillum brasilense* Sp7, and *Pseudomonas putida* KT2440 were routinely grown in minimal saline Dorn medium (Dorn et al., 1974), containing 10 mM fructose, in an orbital shaker (150 rpm) at 30°C. Seeds of *A. thaliana* Col-0, or the mutants when corresponded, were surface sterilized with ethanol 70% for 1 min and then for 7 min with 50% sodium hypochlorite (100% commercial laundry bleach). Afterward, the seeds were rinsed three times with sterile water and kept at 4°C for 4 days to synchronize germination (Poupin et al., 2013). Then, seeds were sown on square Petri dishes with Johnson media (MJ; Cappellari et al., 2019; Finkel et al., 2019) and adjusted at pH 5.7, with agar 1% and supplemented with sucrose 0.5%. Plates were placed vertically in a growth chamber at 22°C with a photoperiod of 16 h of light and 8 h of dark. On day 4 after sowing (4 DAS), seedlings were transplanted to different treatments consisting of the same MJ media (without sucrose) and with or without the inoculation with the respective strain combined with a particular nutritional treatment.

For the phosphate-related treatments, KH_2_PO_4_ (Monobasic potassium phosphate) was used as a source with high solubility (HS-Pi), and Ca_3_(PO_4_)_2_ (Calcium phosphate) as a sparingly soluble source (LS-Pi) that has been used in solubilization experiments (Narang et al., 2000; Poirier and Bucher, 2001; Alzate Zuluaga et al., 2021). In the case of iron, FeSO_4_ (iron(II) sulfate or ferrous sulfate) was used as a highly soluble source (HS-Fe) that contains Fe^2+^. This form is easily absorbed by plants, and Fe_2_(SO_4_)_3_ (iron(III) sulfate or ferric sulfate) as a source with lower solubility (LS-Fe) that contains Fe^3+^ (Morrissey and Guerinot, 2009). This form must be reduced to Fe^2+^ to be uptake by plants (Vert et al., 2003). The different sources of P and Fe were mixed up to reach different concentrations depending on the experiment. Control was considered with 1 mM KH_2_PO_4_ and the low Pi with 0.05 mM KH_2_PO_4_. Control-Fe media had 100 μM FeSO_4_ and deficient media 0.25 μM FeSO_4_. To prepare the inoculated plates, the initial inoculum (10^8^ CFU/ml) was homogenously diluted in the MJ agar just before gelling to reach a final concentration of 10^4^ CFU per ml of medium. For the long-term *in-vitro* experiment, similar conditions of nutritional deficiency of phosphorus and iron were generated. Seedlings at 4 DAS were transplanted to 200 ml sterile glass jars containing MJ media without inoculation (WB) or inoculated with Sp7 or PsJN strains. Also, the nutritional factor was included with the following levels: 1 mM KH_2_PO_4_/100 μM FeSO_4_ (Control), 1 mM Ca_3_(PO_4_)_2_, 0.1 mM Ca_3_(PO_4_)_2_, 25 μM FeSO_4_, 25 μM Fe_2_(SO_4_)_3_ and 0.25 μM Fe_2_(SO_4_)_3_. Data were collected 30 days after transplantation.

### Rhizospheric colonization determination

Eight plants (replicates) per treatment were collected individually in 1.5 ml Eppendorf tubes and vortexed in phosphate buffer for 2 min. Serial dilutions were plated in R2A medium in Petri dishes. Bacteria were counted as colony-forming units (CFU), and data were normalized for each plant using its root area. Contamination was discarded using the non-inoculated plants as control.

### Characterization of the *phr1* and *phl1* mutant genotypes

*Arabidopsis thaliana* Columbia-0 (Col-0) was used as the wild-type genotype in all experiments. Homozygous lines for PHR1 (AT4G28610, SALK_067629 or *phr1-1*) and for PHL1 (AT5G29000, SAIL_B731_B09 or *phl1-1*; and SALK_079505 or *phl1-2*) were isolated. Confirmation by PCR of T-DNA insertion in the target genes was performed using an insertion-specific LB primer in addition to one gene-specific primer. Primers used to genotype the mutant lines were: PHL1-F: TCCCACAATCC AAATTCAGAG, PHL1-R: GTGGAGACGTTT CTGCACTTC, PHR1-F: GACCATTAGGACAAACCTACCA, PHR1-R: TGCATTAGCAGGGAACTAAAGAA, LBb1.3: ATTTTGCCG ATTTCGGAAC, and LB1: GCCTTTTCAGAAATGGATAAATAGCCTTGCTTCC.

### Nutrient analysis in plants

For the nutritional analyses, seeds were sown in MJ media with or without the inoculation of the respective strain in combination with a particular nutritional treatment. Plates were placed horizontally, and the same growth conditions described above were used. At 21 DAS, 210 plants per treatment were collected, pooled, and dried for 48 h at 60°C. The metal content was analyzed in the dried tissues by HNO_3_/HCl block digestion and analysis by inductively coupled plasma optical emission spectrometry (ICP-OES) using the instrument Thermo Scientific iCAP 6,300 Duo (Wheal et al., 2011). Nitrogen was analyzed by the Dumas combustion method using the Leco CN628 instrument.

### Phenotypical and statistical analyses

At the end of each experiment, Petri dishes were scanned (Epson Perfection V600 Photo Scanner), and the images were analyzed using image analysis. The primary root length and rosette areas were calculated using the Image J software (Poupin et al., 2016). The root area and the non-green rosette area were calculated using the Adobe Photoshop software as described in Pinedo et al. (2015). When the experiments considered two factors (bacteria and nutrients), two-way ANOVA was used, followed by a multiple comparisons test. When data was not parametric, Kruskal-Wallis or Mann-Whitney tests were performed to compare subgroups of each data (i.e., comparing bacterial treatments and the non-inoculated group under the same nutritional treatment). The replicate number is indicated in each experiment.

### RNA extraction, cDNA synthesis, and qRT-PCR analyses

Seedlings at 4DAS were transplanted to inoculated plates with the Sp7 or PsJN strains (as described before) or without bacteria (WB). Additionally, plates contained different levels of the nutritional treatment. For P: 1 mM KH_2_PO_4_/0 mM KH_2_PO_4_ or 0.1 mM KH_2_PO_4_/0.99 mM Ca_3_(PO_4_)_2_. For Fe: 25 μM FeSO_4_/0 μM Fe_2_(SO_4_)_3_ or 0.25 μM FeSO_4_/24.75 μM Fe_2_(SO_4_)_3_. Seedlings were collected before being transplanted to the different treatments (control at 0 h) and after 2 h, 24 h, and 7 days (7d) in each treatment. Then, the samples were frozen in liquid nitrogen. RNA was obtained using the Trizol (Invitrogen™, United States) method following the manufacturer’s instructions. For cDNA synthesis, 1 μg of total RNA treated with DNAse I (RQ1, Promega, United States) was reverse transcribed with random hexameric primers using the ImProm II reverse transcriptase (Promega, United States), according to the manufacturer’s instructions. Real-time (RT)-PCR was performed using the Brilliant SYBR Green QPCR Master Reagent Kit (Agilent Technologies, United States) and the Eco RT PCR detection system (Illumina, United States) as described by Poupin et al. (2016). The PCR mixture (10 μl) contained 2.0 μl of template cDNA (diluted 1:10) and 140 nM of each primer. Amplification was performed under the following conditions: 95° C for 10 min, followed by 40 cycles of 94°C, 20 s; 53–64°C, 20 s; and 72°C, 20 s, followed by a melting cycle from 55 to 95°C. Gene expression levels were calibrated using the average value in the samples of non-inoculated plants (WB) in the full P or Fe media (t0). An accurate ratio between the expression of the gene of interest (GOI) and the housekeeping (HK) gene was calculated according to the following equation (Timmermann et al., 2019):


$$\frac{\;{\left(1+E_{GOI}\right)}^{-\left(ct\;GOI-ct\;GOI\;\text{calibrated}\right)}}{{\left(1+E_{HK}\right)}^{-\left(ct\;HK-ct\;HK\;\text{calibrated}\right)}}$$

Efficiency values for each primer set were close to 1, and R2 values were over 99% (Bustin et al., 2009). All experiments were performed in three biological replicates (2 to 5 plants each) and two technical replicates. The expression of three HK genes was analyzed for treatments AtSAND (At2g28390), PP2A (At1g13320), and TIP41-like (At4g34270), using previously described PCR primers (Poupin et al., 2016). In all cases, the expression of HK genes was highly stable, and similar results were obtained using them as normalization genes (Czechowski et al., 2005). Data presented here represent normalization using AtSAND amplification. The PCR primer list is presented in Supplementary Table 1.

## Results

### Plant growth-promoting effects of beneficial proteobacteria in Arabidopsis depend on the interaction between the nutrient source and the strain identity

To evaluate the effects of different Plant Growth-Promoting Bacteria (PGPB) in the growth of Arabidopsis plants exposed to different P and Fe nutritional contexts, three PGPB were used, all belonging to the proteobacteria class. *Paraburkholderia phytofirmans* PsJN (β-proteobacteria), *Azospirillum brasilense* Sp7 (α-proteobacteria) and *Pseudomonas putida* KT2440 (γ-proteobacteria), which represent genera with different described growth-promoting traits (Poupin et al., 2013, 2016; An and Moe, 2016; Cruz-Hernández et al., 2022; Sun et al., 2022). Briefly, *Azospirillum brasilense* Sp7 fixes nitrogen and produces Indol-3-acetic acid (IAA; Compant et al., 2010; Hong et al., 2019). Among other capacities, *Paraburkholderia phytofirmans* PsJN produces siderophores (Esmaeel et al., 2018), synthetizes and degrades IAA (Zúñiga et al., 2013; Naveed et al., 2015; Donoso et al., 2017), and protects plants to different biotic and abiotic stresses (e.g., Pinedo et al., 2015; Ledger et al., 2016; Miotto-Vilanova et al., 2016; Timmermann et al., 2017). *Pseudomonas putida* KT2440 produces IAA (Chea et al., 2021), siderophores (Joshi et al., 2014) and solubilizes phosphate (An and Moe, 2016; Bator et al., 2020; Roslan et al., 2020).

**Figure 1:**
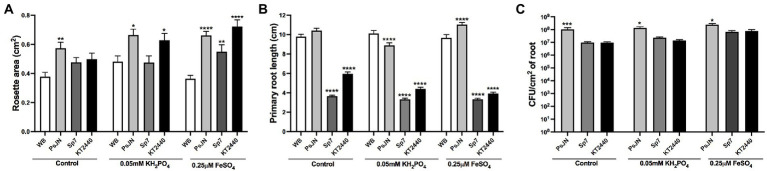
Effects of different phosphate and iron concentrations in plant growth in *Arabidopsis thaliana* inoculated with beneficial rhizobacteria. Rosette area **(A)**, primary root length **(B)**, and root colonization **(C)** in plants 15 days after sowing in control medium (1 mM KH_2_PO_4_/100 μM FeSO_4_), low-P (0.05 mM KH_2_PO_4_/100 μM FeSO_4_), or low-Fe (1 mM KH_2_PO_4_/0.25 μM FeSO_4_). Non-inoculated plants are noted as WB, and inoculations with *Paraburkholderia phytofirmans* PsJN, *Azospirillum brasilense* Sp7, or *Pseudomonas putida* KT2440 are noted as PsJN, Sp7, and KT2440, respectively. Data are means ± SE of 18 replicates per treatment. Asterisks indicate significant differences between each treatment and the control group according to a Two-way ANOVA with multiple comparisons test (^*^p < 0.05; ^**^p < 0.01; ^***^p< 0.001; and ^****^p < 0.0001).

First, the effects of these strains were evaluated in plants exposed to Pi or Fe deficiency. Control plants were grown in 1 mM KH_2_PO_4_ and 100 μM FeSO_4_ (control), low-Pi was assessed at 0.05 mM KH_2_PO_4_ and low-Fe at 0.25 μM FeSO_4_. PsJN strain increased the rosette area in all the treatments (Figure 1A). The other strains affected the rosette area depending on the nutritional treatment. For instance, in Fe deficiency, KT2440 doubled the rosette area compared to the non-inoculated plants in the same treatment (Figure 1A). This strain also increased the rosette area in low-Pi, but not in the control group. Sp7 and KT2440 strains decreased the primary root length (around 39% to 65% less) regardless of the treatment, while PsJN had minor effects (Figure 1B). All strains were able to colonize roots regardless of the nutritional treatments. Nevertheless, the PsJN strain had higher colonization levels with a mean of around 10^8^ CFU/cm^2^ of root compared to Sp7 and KT2440, which reached levels from 10^6^ to 10^7^ CFU/cm^2^ of root irrespective of the nutritional treatment (Figure 1C).

**Figure 2:**
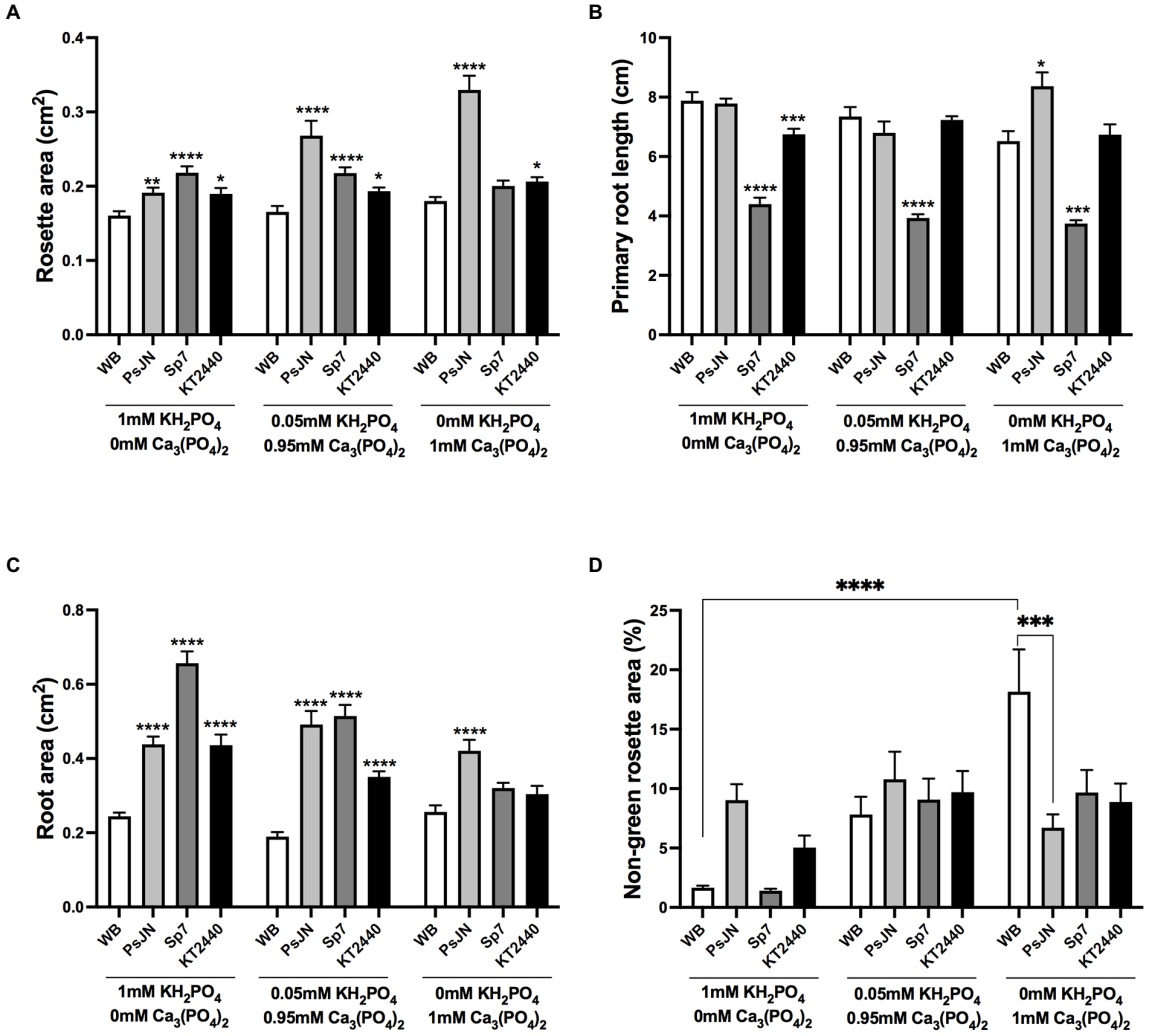
Effects of different sources of phosphorus in plant growth and stress parameters in *Arabidopsis thaliana* inoculated with beneficial rhizobacteria. Rosette area **(A)**, primary root length **(B)**, root area **(C)**, and non-green rosette area **(D)** in plants 15 days after sowing in media with different concentrations of KH_2_PO_4_ or Ca_3_(PO_4_) as a phosphate source with high or low solubility, respectively (Factor 1). A second factor corresponds to the inoculation, where non-inoculated plants are noted as WB, and inoculations with *Paraburkholderia phytofirmans* PsJN, *Azospirillum brasilense* Sp7, or *Pseudomonas putida* KT2440 are noted as PsJN, Sp7, and KT2440, respectively. Data are means ± SE of 30 replicates per treatment. In the case of A and B panels, asterisks indicate significant differences between each treatment and their respective WB treatment (Kruskal–Wallis test, ^*^p < 0.05; ^**^p < 0.01; ^***^p< 0.001; and ^****^p < 0.0001). For panel C, only some significant differences are indicated with lines as asterisks, according to a Two-Way ANOVA with multiple comparisons ^*^p < 0.05; ^**^p < 0.01; ^***^p < 0.001; and ^****^p < 0.0001.

Some soils present sufficient quantities of phosphate, but the element is present in chemical sources with low bioavailability. Then, two phosphate sources with different solubilities were evaluated in the plant-beneficial bacteria interaction. KH_2_PO_4_ (monobasic potassium phosphate) was used as a source with high solubility (HS-Pi), and Ca_3_(PO_4_)_2_ (calcium phosphate) as a sparingly soluble source (LS-Pi; Narang et al., 2000; Poirier and Bucher, 2001). Both sources were mixed up considering the control situation at a final concentration of 1 mM, and the same PGPB strains described above were used as the inoculation factor. No significant changes were observed in the non-inoculated (WB) plants in the rosette area (Figure 2A) or primary root length (Figure 2B), but a significant increase (16.5%) in the non-green area was observed when plants were grown in 1 mM LS-Pi compared to 1 mM HS-PI (Figure 2D). PsJN-inoculated plants grown in LS-Pi and inoculated with PsJN showed the largest rosettes, which were significantly different from the non-inoculated plants in the same P treatment. Also, these plants showed an increase of 78% compared to those grown in 1 mM HS-Pi and inoculated with the same strain (Figure 2A). KT2440 strain induced a minor increase in rosette areas irrespective of the P source. Remarkably, the Sp7 strain lost its promoting effect when P was supplied in a low solubility source (Figure 2A). KT2440 and PsJN strains had none or modest effects in the primary root length, independently on the P source (Figure 2B). On the other hand, Sp7 strain inoculation reduced the length of the primary roots independently on the P source (Figure 2B). PsJN increased the root area in a range of 75 to 104% compared with to the non-inoculated plants independently on the P source (Figure 2C), while the Sp7 strain induced the highest effect in this parameter when plants were grown in HS-Pi. Interestingly, both Sp7 and KT2440 strains lost their effects in the root area when plants were grown in LS-Pi as the sole P source but no when a small amount of HS-Pi (0.05 mM) was added to the media (Figure 2C). Regarding the non-green area in plants growing in low solubility P, all strains significantly reduced this stress parameter compared with non-inoculated plants, with the highest reduction (63%) induced by PsJN strain inoculation (Figure 2D).

**Figure 3:**
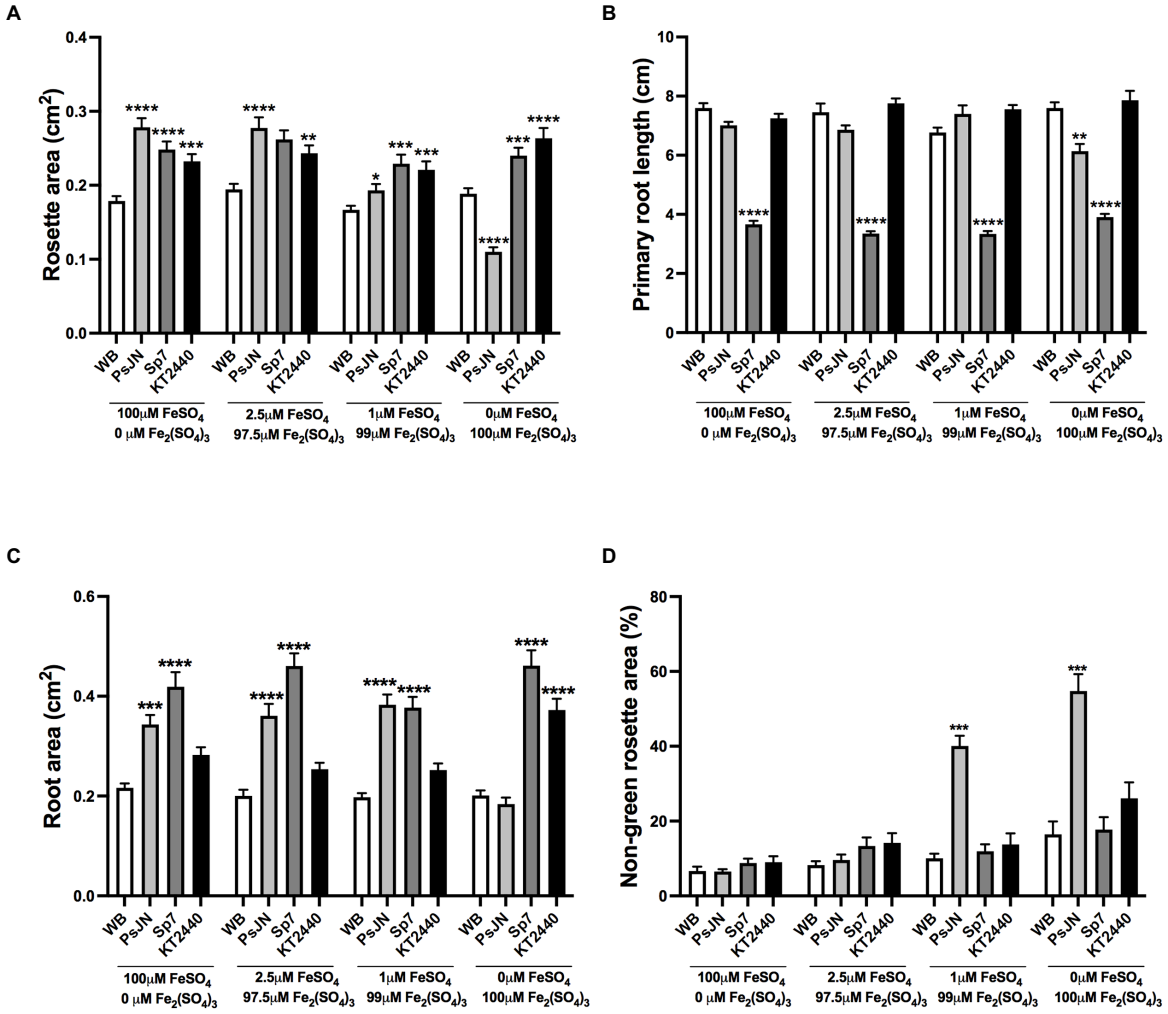
Effects of different sources of iron in plant growth and stress parameters in *Arabidopsis thaliana* inoculated with beneficial rhizobacteria. Rosette area **(A)**, primary root length **(B)**, root area **(C)**, and non-green rosette area **(D)** in plants 15 days after sowing in media with different concentrations of FeSO_4_ or Fe_2_(SO_4_)_3_, as an iron source with high or low solubility, respectively (Factor 1). A second factor corresponds to the inoculation, where non-inoculated plants are noted as WB, and inoculations with *Paraburkholderia phytofirmans* PsJN, *Azospirillum brasilense* Sp7, or *Pseudomonas putida* KT2440 are noted as PsJN, Sp7, and KT2440, respectively. Data are means ± SE of 30 replicates per treatment. Asterisks indicate significant differences between each treatment and their respective WB treatment (Kruskal–Wallis test, ^*^p < 0.05; ^**^p < 0.01; ^***^p< 0.001; and ^****^p < 0.0001).

In terms of the source of Fe, a similar experiment was performed, using FeSO_4_ (iron(II) sulfate or ferrous sulfate) as a highly soluble source (HS-Fe) that contains Fe^2+^, the form that is absorbed by non-grasses plants (Morrissey and Guerinot, 2009), and Fe_2_(SO_4_)_3_ (iron(III) sulfate or ferric sulfate) as a source with lower solubility (LS-Fe) that contains Fe^3+^ a form that has to be reduced to be uptake as Fe^2+^ (Vert et al., 2003). In this case, no significant differences were observed in the rosette area (Figure 3A), primary root length (Figure 3B), root area (Figure 3C), or non-green area (Figure 3D) in the non-inoculated plants independent of the Fe source used. Interestingly, contrary to Sp7 or KT2440 strains, which increased the rosette areas independent of the Fe treatment, PsJN showed positive effects only when FeSO_4_ was present but had negative effects when Fe was delivered exclusively as Fe_2_(SO_4_)_3_, reducing the rosette area in a 44% compared to the non-inoculated plants in the same treatment (Figure 3A; *p* < 0.0001). Similar to what was observed in the phosphate experiments, KT2440 or PsJN produced little or no change in the primary root length independent of the Fe treatment. However, Sp7-inoculated plants showed a reduction in the primary root length independently of the iron source (Figure 3B). In the case of the root area, an interaction effect was detected between the inoculation and Fe-source (Two-way ANOVA, 12.21% of the total variance, *p* < 0.0001). The inoculation also had a significant difference, accounting for 32.94% of the total variance (Two-way ANOVA, *p* < 0.0001). Specifically, the Sp7 strain increased the root area in all the treatments, and PsJN lost its effect when Fe was delivered exclusively as LS-Fe. The KT2440 strain only had a significant effect in the latter condition (Figure 3C). Moreover, the rosette areas of PsJN inoculated plants showed higher proportions of non-green areas (up to 4 times) in 1 μM or 0 μM FeSO_4_ (Figure 3D).

**Figure 4:**
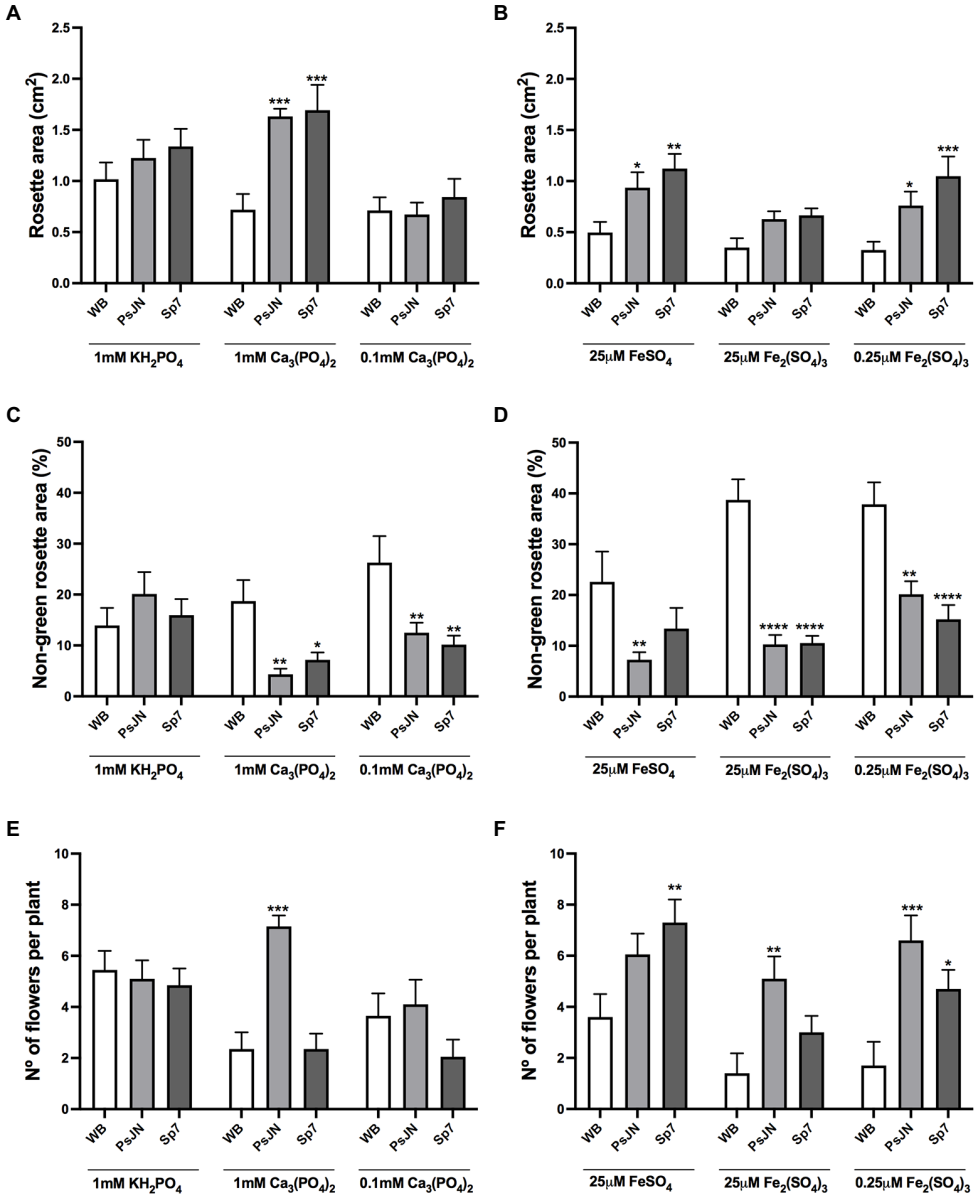
Long-term effects of different sources of phosphorus and iron in plant growth and flowering in *Arabidopsis thaliana* inoculated with beneficial rhizobacteria. Rosette area **(A,B)**, non-green rosette area **(C,D)**, and the number of flowers per plant **(E,F)** in plants 34 days after sowing. Factor 1 consisted of different concentrations of KH_2_PO_4_/Ca_3_PO_4_ (as phosphate sources with different solubilities; **A,C,E**) or FeSO_4_/Fe_2_(SO_4_)_3_ (as iron sources with different solubilities; **B,D,F**). Factor 2 consisted of non-inoculated plants (WB) or inoculated with *Paraburkholderia phytofirmans* PsJN (PsJN) or *Azospirillum brasilense* Sp7 (Sp7). Data are means ± SE of 20 replicates per treatment. Asterisks indicate significant differences between each treatment and their respective WB treatment (Two-way ANOVA and Tukey multiple comparisons, ^*^p < 0.05; ^**^p < 0.01; ^***^p< 0.001, and ^****^p < 0.0001).

To evaluate the long-term effects over time of different sources of P and Fe, together with the inoculation with beneficial bacteria, plants were grown with 1 mM HS-Pi, 1 mM LS-Pi, or 0.1 mM LS-Pi (Figures 4A,C,E). Also, with 25 μM HS-Fe, 25 μM LS-Fe, and 0.25 μM LS-Fe (Figures 4B,D,F). Inoculation was performed with PsJN or Sp7 strains as they showed contrasting effects in the previous experiments. After 34 days, no significant differences were observed in the inoculated plants grown in HS-Pi (1 mM KH_2_PO_4_, Figure 4A) or low concentrations of LS-Pi (0.1 mM Ca_3_PO_4_). Instead, larger rosettes were promoted by inoculation with any of the strains at 1 mM LS-Pi (Figure 4A). When the levels and sources of Fe were changed, the plants showed smaller rosettes areas than in the control treatment (Figure 4A). Nevertheless, both strains promoted larger rosette areas compared to the non-inoculated plants regardless of the nutritional factor (Figure 4B). The non-green rosette areas were bigger in the treatments with LS-Fe (Figure 4D) than those with different sources of P (Figure 4C). Both strains were able to reduce this stress proxy in almost all the nutritional conditions (Figures 4C,D).

The PsJN strain accelerated flowering only when 1 mM LS-Pi was used (Figure 4E; Two-way ANOVA, Tukey’s multiple comparisons; *p* < 0.0001). Interestingly, Sp7 did not induce changes in flowering time in the different P treatments (Figure 4E). When the Fe supply was altered, PsJN maintained its ability to induce flowering independently on the Fe treatment (Figure 4F; Two-way ANOVA, Tukey’s multiple comparisons; *p* < 0.0005) and Sp7 only accelerated flowering in the low-Fe treatment (Figure 4F).

**Figure 5:**
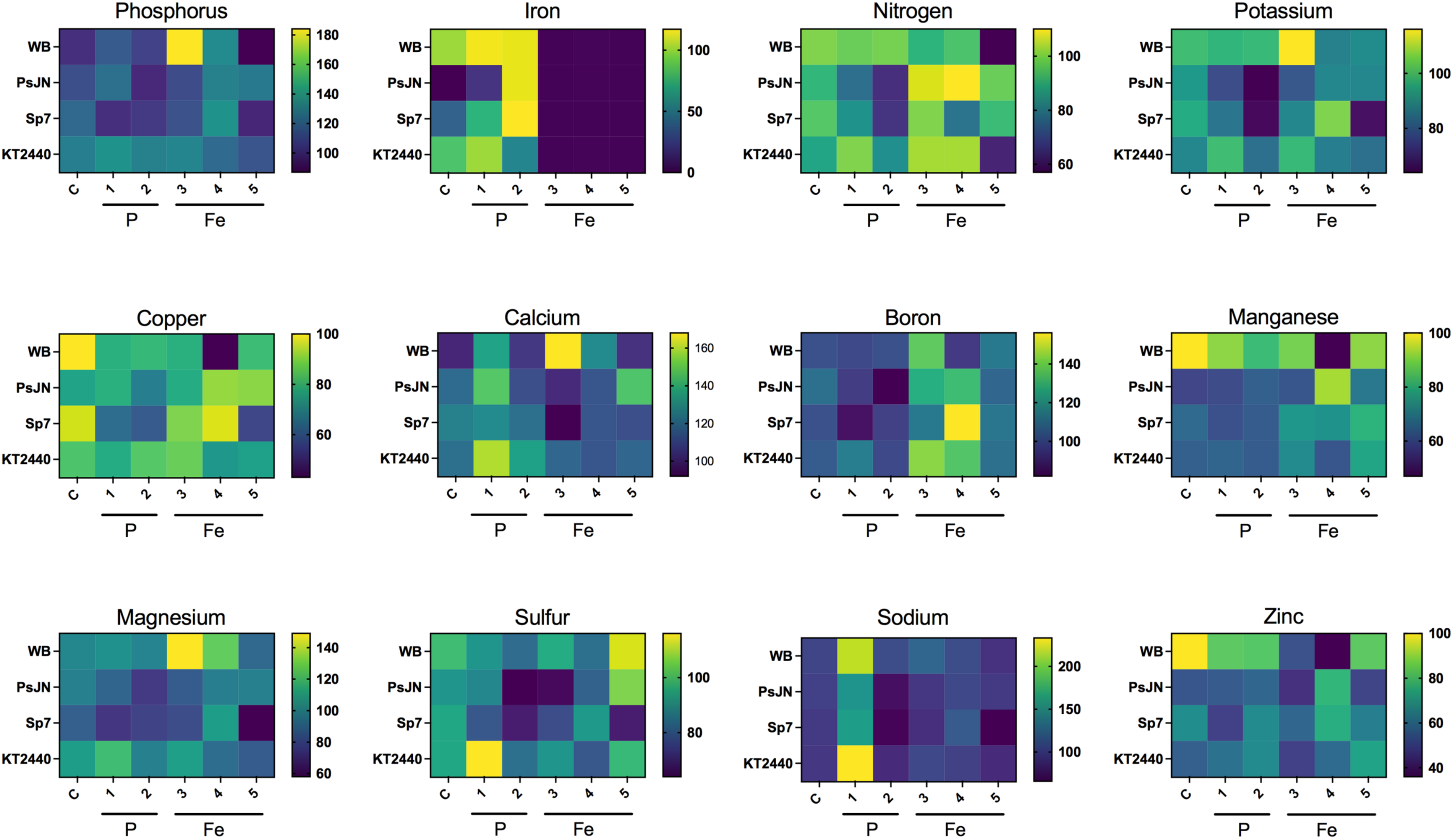
Effects on the nutrient content of *Arabidopsis thaliana* plants of different sources of phosphorus and iron and the inoculation with beneficial rhizobacteria. The nutritional treatments are indicated in the X-axis of the figures, being (C) the full culture media, 1 mM KH_2_PO_4_ and 100 μM FeSO_4_; (1) 0.05 mM KH_2_PO_4_/0.95 mM Ca_3_PO_4_; (2) 0.05 mM KH_2_PO_4_; (3) 25 μM FeSO_4_; (4) 0.25 μM FeSO_4_/24.75 μM, and (5) 0.25 μM FeSO_4_. The Y-axis represents the inoculation treatments, with (WB) without bacteria; (PsJN) *Paraburkholderia phytofirmans* PsJN; (Sp7) *Azospirillum brasilense* Sp7 (Sp7), and (KT2440) *Pseudomonas putida* KT2440. Each square represents data relative to the C/WB treatment (100%). The color from yellow to blue indicates high to low nutrient levels.

The nutrient content of plants at 21 DAS (as an intermediate time between the short and long-term experiments) was analyzed in dried aerial and root tissues of plants exposed to the different treatments (inoculation x nutrition combinations; Figure 5). For phosphorus, changes in content depended on the nutritional state and inoculation factors. Compared to the non-inoculated plants, KT2440 induced the most notorious increases in this element, independent of the treatment (up to 36%), and was the only strain that increased this nutrient in the low-Pi treated plants (29% more than the non-inoculated plants in the same treatment; Figure 5). Regarding the iron content, the nutritional treatment had severe effects. Plants grown under low iron concentrations (25 μM of Fe or less) presented ~1% of the iron in the control plants. In most cases, inoculated plants presented less iron content than the non-inoculated plants in the same nutritional treatments. However, in the 0.25 μM FeSO_4_ treatment, the inoculated plants presented no significant differences from the non-inoculated ones (Figure 5). An interaction between Pi and Fe was detected, where plants treated with low P presented ~10% more Fe than the control plants (excepting the KT2440-inoculated plants that presented a 47% less). Regarding other nutrients, in some cases the inoculation factor seems to have a higher effect than the nutritional factor (i.e., manganese and zinc), regardless of the strain (Figure 5). In other cases, such as nitrogen, potassium, and calcium, the inoculation effect changed depending on the nutritional treatment (Figure 5). In magnesium, sulfur, copper, and sodium there was no clear effect of either of the two factors or their interaction (Figure 5). Interestingly, in the low-Fe treatment the non-inoculated plants presented 57% of the nitrogen compared to control non-inoculated plants (Figure 5). In the same scenario, PsJN and Sp7-inoculated plants presented 98 and 93% compared to the same control treatment, respectively (Figure 5). However, in the low-Pi treatment the non-inoculated plants had similar amounts to the control (99%), but the PsJN and Sp7-inoculated plants had 64% and 65%, respectively (Figure 5). As was observed in the case of iron, boron contents changed drastically depending on the nutritional treatments, being increased in the treatment with low-Fe (Figure 5).

### The nutritional context influences the rhizobacterial modulation of different genetic pathways in Arabidopsis

**Figure 6:**
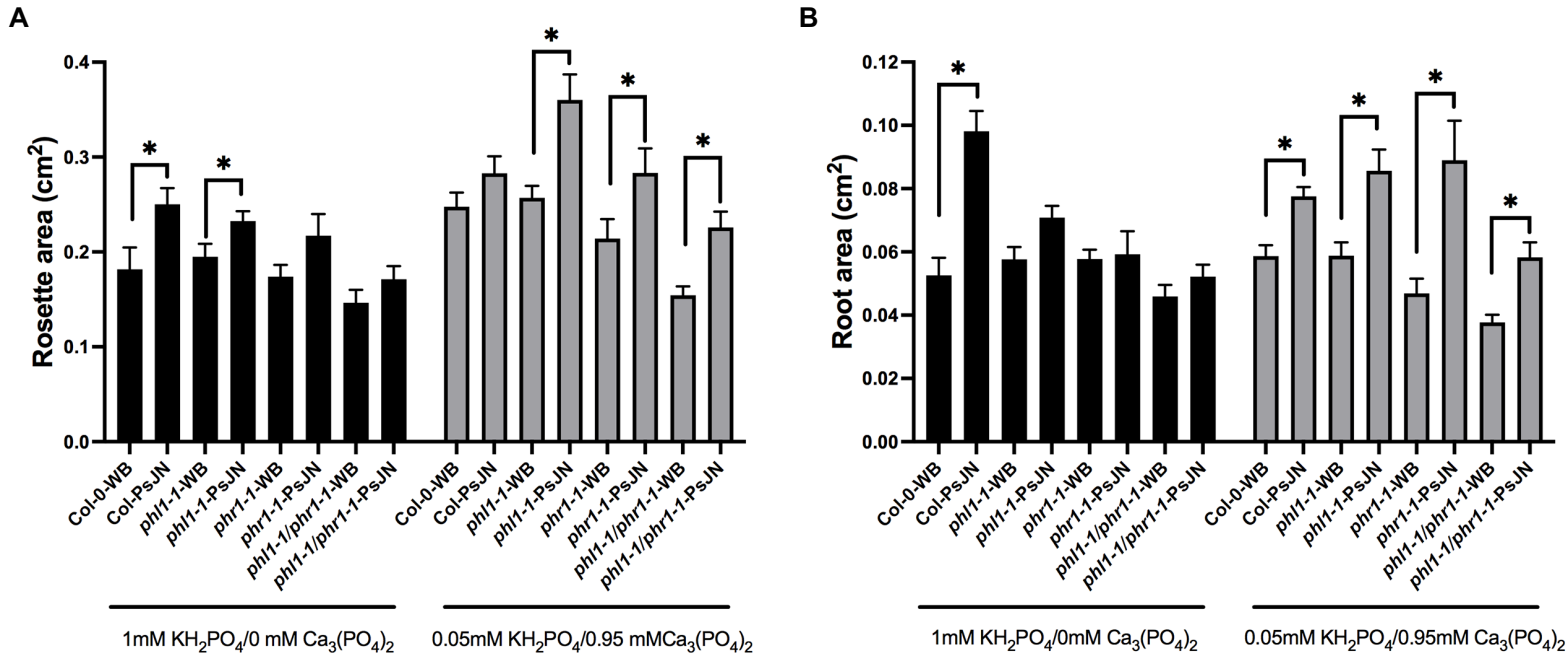
Effects of different sources of phosphorus and inoculation with beneficial rhizobacteria in the growth of *phr1 and phl1 Arabidopsis thaliana* mutants in phosphate starvation response. Rosette **(A)** and root area **(B)** in plants 15 days after sowing. Factor 1 consists of different concentrations of KH_2_PO_4_/Ca_3_PO_4_ (as phosphate sources with different solubilities). Factor 2 consists of non-inoculated plants (WB) or inoculated with *Paraburkholderia phytofirmans* PsJN (PsJN). Factor 3 consists of the genotype of Arabidopsis plants (Col-0; the single mutants *phl1-1* or *phr1-1* and the double mutant *phl1/phr1-1*). Asterisks indicate significant differences in paired comparison for each genotype (WB versus PsJN inoculation) according to a non-parametric Mann-Whitney test (*p < 0.05).

Under phosphate starvation, two partially redundant transcription factors regulate the physiological responses in Arabidopsis, PHOSPHATE STARVATION RESPONSE 1 (PHR1) and PHR1-LIKE (PHL1; Bustos et al., 2010). Concordantly, the double mutant *phr1 phl1* has an impaired PSR and accumulates a low level of Pi (Finkel et al., 2019). Here we tested the effects of the interaction between different sources of Pi and the strain PsJN in the single mutants *phl1-1* and *phr1-1*, as also in the double mutant. Regarding the aerial zone, different results were obtained depending on the Pi source (Figure 6A). When only HS-Pi was supplied, the PsJN strain significantly induced the rosette growth in the wild-type plants and the *phl1-1* mutant, but not in the *phr1-1* or the double mutant (Figure 6A). Instead, when Pi was delivered mostly as LS-Pi, PsJN had greater effects in the three mutant genotypes (Figure 6A). Even more drastic differences were observed in the root area (Figure 6B), where PsJN strain did not induce any change in the mutants when the Pi source was highly soluble. On the other hand, PsJN induced significant increases in the root area both in the wild-type and mutant ecotypes when the Pi was mainly LS-Pi (Figure 6B). To discard that the effects were related to different colonization capacities of the strain in the different ecotypes, the colonization was monitored by plate counting as described in the material and method section. Colonization was detected in the roots of plants in all the treatments and genotypes in ratios from 10^7^ufc/cm to 10^8^ufc/cm^2^ of root.

**Figure 7:**
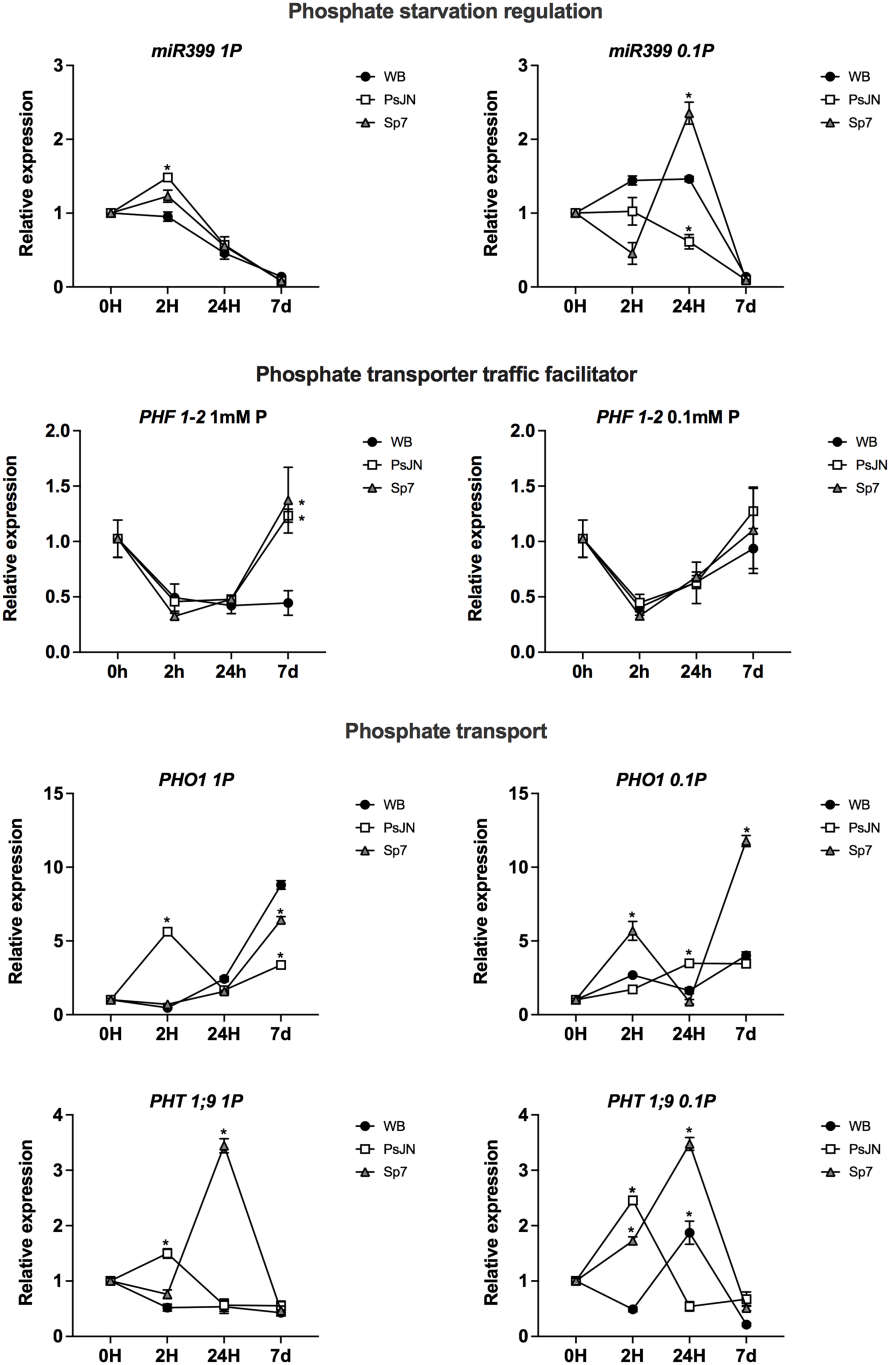
Effects of beneficial bacteria on phosphate response-related genes in *Arabidopsis thaliana* grown in different phosphate sources. Quantitative RT-PCR determinations of relative expression levels of the genes: *PHR1-1 (PHOSPHATE STARVATION RESPONSE 1)*; *PHL (PHR1-LIKE 1); PHF1-2 (PHOSPHATE TRANSPORTER TRAFFIC FACILITATOR 1)*. Plants were exposed to 1 mm KH_2_PO_4_/0 mM Ca_3_PO_4_ (Left panel) or 0.1 mm KH_2_PO_4_/0.99 mM Ca_3_PO_4_ (right panel) for 0, 2, 24 h or 7 days (d). Simultaneously, plants were non-inoculated (WB) or inoculated with *Paraburkholderia phytofirmans* PsJN (PsJN) or *Azospirillum brasilense* Sp7 (Sp7). Normalization was performed with the housekeeping *SAND* family gene (AT2G28390). Asterisks indicate statistical significance among treatments in a particular time compared to the WB group (Two-way ANOVA and multiple comparisons, *p* < 0.05).

The transcriptional profile of several genes related to PSR or different hormonal pathways was analyzed in plants exposed to the same experimental Pi schemes used before. RNA was extracted at 0, 2, 24 h, and 7d after the concomitant exposure to different Pi sources and the PsJN and Sp7 strains. No significant differences were observed in *PHR1-1* or *PHL1-2* regarding the Pi source or the inoculation with both rhizobacteria (Supplementary Figure 1). In all the treatments, the transcripts presented the highest upregulation at 7d (Supplementary Figure 1). The PSR is also regulated by the microRNA *miR399*, which is upregulated in low-Pi conditions and downregulates *PHO2* (Bari et al., 2006). PHO2 is an E2-UBC that negatively affects shoot phosphate content mediating the degradation of PHF1 (PHOSPHATE TRANSPORTER TRAFFIC FACILITATOR1) which is required for membrane localization of high-affinity phosphate transporters, allowing Pi transport into roots (González et al., 2005). *miR399* transcript was differentially regulated by Pi-source, its highest accumulation was induced by the Sp7 strain after 24 h in 0.1 mm HS-Pi/0.99 mM LS-Pi (Figure 7). Compared to the non-inoculated plants, the PsJN strain upregulated *miR399* 2 h in 1 mM HS-Pi, but downregulated it 2 and 24H in 1 mm HS-Pi/0.99 mM LS-Pi (Figure 7). On the other hand, *PHO2* did not show a significant regulation by the P treatment or the inoculation (Supplementary Figure 1). Interestingly, an upregulation of *PHF1* was detected by PsJN and Sp7 strains, but only after 7 days in the highly soluble Pi source (Figure 7, left panel). The phosphate transporter PHO1 (ARABIDOPSIS PHOSPHATE 1) and PHT1;9 (PHOSPHATE TRANSPORTER 1;9) are both involved in P translocation from the roots to the shoots (Pegler et al., 2020; Wang et al., 2021). In the case of *PHO1* transcript, the effect of the inoculation changed depending on the Pi source, with its highest upregulation by Sp7 after 7d in 0.1 mm HS-Pi/0.99 mM LS-Pi (Figure 7). Similarly, *PHT1;9* was highly upregulated by the Sp7 strain after 24 h, regardless of P treatment (Figure 7). Other P transporters coding genes, such as *PHT1;1* and *PHT1;4* were not significantly regulated by the treatments (Supplementary Figure 1).

**Figure 8:**
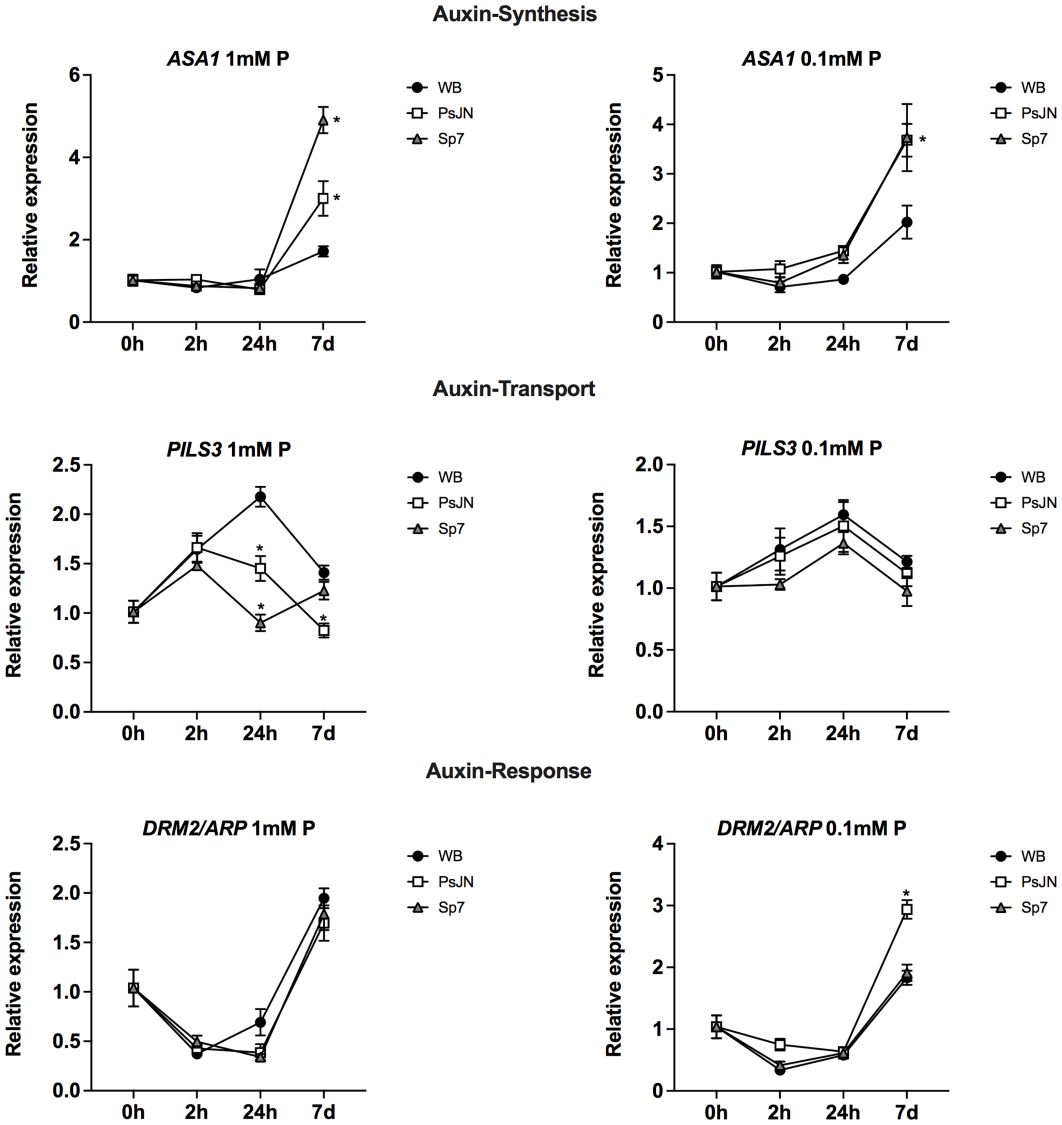
Effects of beneficial bacteria on auxin-related genes in *Arabidopsis thaliana* grown in different phosphate sources. Quantitative RT-PCR determinations of relative expression levels of the genes related to auxin synthesis *ASA1* (*ANTHRANILATE SYNTHASE ALPHA SUBUNIT 1*); transport *PILS3* (*PIN-LIKES 3*), and response *DRM2/ARP1* (*DORMANCY ASSOCIATED GENE-1/AUXIN-REPRESSED PROTEIN*). Plants were exposed to 1 mm KH_2_PO_4_/0 mM Ca_3_PO_4_ (Left panel) or 0.1 mm KH_2_PO_4_/0.99 mM Ca_3_PO_4_ (right panel) for 0, 2, 24 h or 7 days (d). Simultaneously, plants were non-inoculated (WB) or inoculated with *Paraburkholderia phytofirmans* PsJN (PsJN) or *Azospirillum brasilense* Sp7 (Sp7). Normalization was performed with the housekeeping *SAND* family gene (AT2G28390). Asterisks indicate statistical significance among treatments in a particular time compared to the WB group (Two-way ANOVA and multiple comparisons, *p* < 0.05).

Rhizobacteria can also promote growth by producing different phytohormones or by modulating their signaling pathways, as in the case of the PsJN strain (Poupin et al., 2013, 2016). Then, the gene regulation of several genes related to auxin (Figure 8) and ethylene/jasmonic acid (Supplementary Figure 2) was analyzed. *ASA1* (*ANTHRANILATE SYNTHASE ALPHA SUBUNIT 1*) catalyzes the first step of tryptophan (Trp) biosynthesis, which is a precursor of auxin biosynthesis (Mano and Nemoto, 2012). According to a Two-Way ANOVA Analysis, in both Pi treatments, there was an interaction effect (inoculation x time, *p* < 0.005), with an increased transcript accumulation at 7d. Also, the inoculation had a significant effect, where both strains upregulated the gene at 7d, compared to their respective non-inoculated treatment (Figure 8). The Sp7 strain induced the highest effects and the PsJN effects were increased in LS-Pi. Still, this difference was not observed with the PsJN strain when 0.1 mM Pi (soluble source) was used (Figure 8, right panel). PILS3 (AT1G76520) is an auxin efflux carrier putatively involved in auxin transport (Barbez et al., 2012), which downregulation in Arabidopsis by PsJN strain was previously reported (Poupin et al., 2013, 2016). Here, different patterns of regulation were observed depending on the Pi source. Changes induced by the strains were only significant when 1 mM HS-Pi was used (*p* < 0.0001), with a downregulation induced by PsJN 24 h that was maintained at 7d (Figure 8, left panel). In addition, as an example of an auxin-response gene, the regulation of *DRM2/ARP, DORMANCY ASSOCIATED GENE-1/AUXIN-REPRESSED PROTEIN* was analyzed as this gene was reported as upregulated by strain PsJN (Poupin et al., 2016). Interestingly, this upregulation was only observed after 7d of treatment with the LS-Pi source (Figure 8, right panel; *p* < 0.0001).

In the case of ethylene (*PDF1.2*) and jasmonic acid (*LOX2*) related genes, an upregulation was observed in both genes 7d after the treatment with Sp7 and PsJN strains, regardless of the treatment with different Pi sources (Supplementary Figure 2).

**Figure 9:**
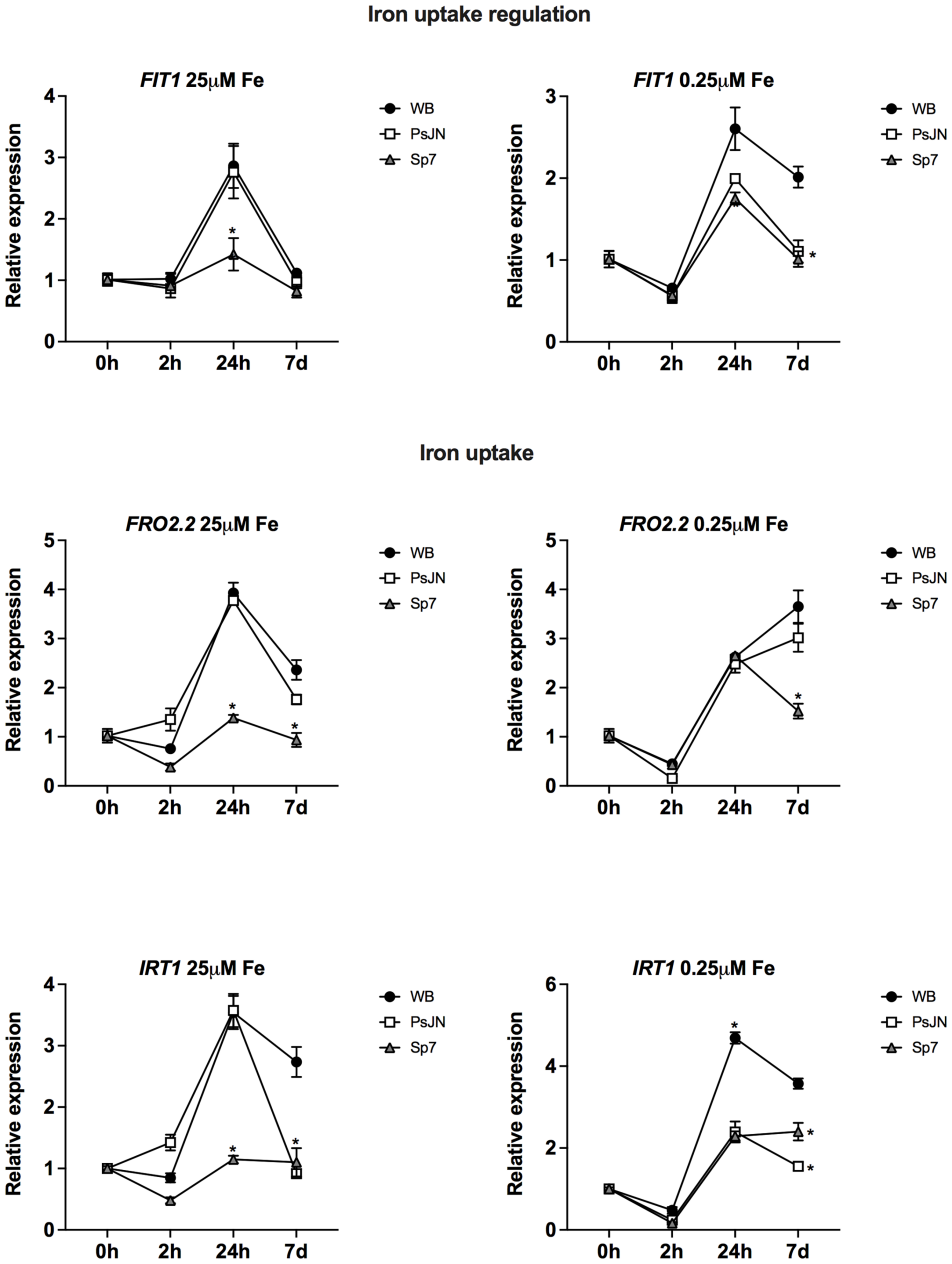
Effects of beneficial bacteria on iron uptake-related genes in *Arabidopsis thaliana* grown in different phosphate sources. Quantitative RT-PCR determinations of relative expression levels of the genes related to iron uptake regulation *FIT1* (*ARABIDOPSIS FE-DEFICIENCY INDUCED TRANSCRIPTION FACTOR 1*); and uptake *FRO2.2* (*FERRIC REDUCTASE OXIDASE 2.2*) and *IRT1* (*IRON-REGULATED TRANSPORTER 1*). Plants were exposed to 25 μM FeSO_4_/0 μM Fe_2_(SO_4_)_3_ as a highly soluble iron source (left panel) or 0.25 μM FeSO_4_/24.75 μM Fe_2_(SO_4_)_3_ as an iron source with low solubility (right panel) for 0, 2, 24 h or 7 days (d). Simultaneously, plants were non-inoculated (WB) or inoculated with *Paraburkholderia phytofirmans* PsJN (PsJN) or *Azospirillum brasilense* Sp7 (Sp7). Normalization was performed with the housekeeping *SAND* family gene (AT2G28390). Asterisks indicate statistical significance among treatments in a particular time compared to the WB group (Two-way ANOVA and multiple comparisons, *p* < 0.05).

Finally, the transcriptional profiles of 3 genes related to iron uptake and uptake regulation were evaluated. In this case, the nutrient factor was affected by exposing the plants to 25 μM HS-Fe/0 μM LS-Fe as a highly soluble iron source (Figure 9, left panel) or 0.25 μM HS-Fe/0 μM 24.75 LS-Fe as an iron source with low solubility. *FIT1* (*ARABIDOPSIS FE-DEFICIENCY INDUCED TRANSCRIPTION FACTOR*) was downregulated by Sp7 at 24 h in the highly soluble Fe form, coincidently with the downregulation of *FRO2.2* and *IRT1* at the same time and treatment (Figure 9). Interestingly, *IRT1* (*IRON-REGULATED TRANSPORTER 1*) was downregulated by both bacteria at 7d, irrespective of the iron treatment (Two-way ANOVA, multiple comparisons, *p* < 0.0001).

## Discussion

Rhizosphere nutrient availability directly regulates plant growth and, in turn, is influenced by many interacting factors such as the physicochemical soil properties, climate, and the microorganisms thriving there (Liu et al., 2022). Rhizospheric microorganisms can modulate the nutrient uptake rate by plants, as also their mobilization/immobilization, release from organic sources, and availability (Darrah, 1993; Liu et al., 2022). In addition, rhizobacteria can promote growth by producing different phytohormones or by modulating their signaling pathways, as in the case of the PsJN strain (Poupin et al., 2013, 2016). In other cases, strains such as *Azospirillum brasilense* Sp7 can produce auxin by themselves (Somers et al., 2005). The last is relevant, considering that the nutritional status of plants has also been linked to different hormonal pathways (Bhosale et al., 2018; López-Ruiz et al., 2020; Yi et al., 2021). The effects of different rhizobacteria on the nutritional status of plants have been reported (Israr et al., 2016; Naqqash et al., 2016; Korir et al., 2017). For instance, Alzate Zuluaga et al. (2021) studied the effects of an *Enterobacter* strain in Maize and Cucumber plants growing in different sources of Pi and found a differential response depending on the plant species. Under low-Pi, the bacterium in combination with Ca_3_(PO_4_)_2_ induced a more remarkable effect on the root architecture of cucumber than in maize (Alzate Zuluaga et al., 2021). Chea et al. (2021) reported the effects of a synthetic community of five PGPR, including *Azospirillum* sp. and *Pseudomonas putida* KT2440, in potato plants under different P levels. They found that under Pi deficiency, the inoculated plants showed root and shoot biomass increase compared to the non-inoculated plants (Chea et al., 2021). Interestingly, these changes were not observed when Pi was present in normal amounts (Chea et al., 2021). Here, the influence on the rosette area of the three strains was affected by the Pi concentration and the solubility of the chemical sources (refer Graphical abstract). For instance, PsJN and KT2440 induced the highest effects in P deficiency (Figure 1A; refer Graphical abstract, A). Comparing different P sources, PsJN induced the highest effects in the treatment with low solubility P (Figure 2A; refer Graphical abstract, A). This correlates with an increase of 23% in P in PsJN-inoculated plants grown in 1 mM LS-Pi compared to the control plants (Figure 5; refer Graphical abstract, C). In the case of Fe, all the strains increased the rosette areas in Fe deficiency with soluble Fe (Figure 1A; refer Graphical abstract, A). Nonetheless, the PsJN strain did not increase this parameter when Fe was delivered only as LS-Fe (Figure 3A). Then, in the short-term, the effects of PsJN strain in the aerial zone depended on the Fe source. Interestingly, this relation was not detected in a long-term experiment, which might indicate an adaptation that requires time to be established.

Liu et al. (2022) found that the increase in microbial nitrogen fixation and N-cycling-related enzyme activities in the rhizosphere led to a 10% increase in available N relative to bulk soil (Liu et al., 2022). However, N fixation has large P requirements, and the need is satisfied only at higher levels of this nutrient (Graham and Vance, 2000; Schütz et al., 2018). *Azospirillum brasilense* is a free-living nitrogen-fixing bacterium, and different strains of this genus have been used to replace N-fertilizers when associated with a variety of crops worldwide (reviewed in Fukami et al., 2018). Nevertheless, the real contribution of nitrogen transfer from this strain to plants is still under debate. Other bacterial traits, such as auxin production, have been related to its growth promotion effects in plants (Spaepen et al., 2014). Here, Sp7 induced an increase in the rosette areas of plants grown in control conditions (Figures 2A, 3A) and in Fe deficiency (Figure 1A). However, this pattern was lost in P deficiency (Figure 1A; refer Graphical abstract, A) or when the Pi was delivered in a low solubility source (Figure 2A; refer Graphical abstract, C). These results could be related to a minor capacity to fix nitrogen under P deficiency. Furthermore, N levels in Sp7 inoculated plants were similar to non-inoculated plants in the control treatment but were reduced to 65% in the low-Pi treatment (Figure 5). As a note of caution to this interpretation, PsJN strain, which does not fix nitrogen, also reduced the levels of N in plants under P deficiency (Figure 5).

Regarding the root system, a limitation or even increased growth has been described under Pi scarcity (Tiziani et al., 2020). KT2440 and Sp7 have been described as auxin producers, a capacity related to the shortening of the primary root in the interacting plants (Spaepen et al., 2014; Schütz et al., 2018). Here, both KT2440 and Sp7 strains decreased the primary root length independently of the P or Fe source and quantity. Then, this capacity seems unrelated to the P and Fe nutritional context (refer Graphical abstract). A larger root surface is related to a better capacity to explore greater soil volume and better nutrient uptake in plants (Wissuwa et al., 2005). Sp7 and KT2440 strains lost their positive effects increasing the root surface in LS-Pi, and PsJN lost its effects in LS-Fe. Thus, the effects of these bacteria in the aerial and root zone are highly dependent on the nutritional context in a species-specific manner. PsJN strain has been described as a strain able to accelerate flowering in Arabidopsis (Poupin et al., 2013). Here, this pattern was also observed, especially in LS-Pi and when the Fe concentrations were diminished (Figures 4E,F).

Complex interactions have been described among nutrients in plants (Lay-Pruitt et al., 2022). Interesting results were obtained in the nutritional analysis of plants. For instance, P content was relatively similar among treatments which is in accordance with the highly regulated mechanisms of P homeostasis that have been reported in plants (Gransee and Merbach, 2000). Still, some differences were induced by the nutritional treatment (P and Fe) and the inoculation factor, where KT2440-inoculated plants presented the highest P levels (Figure 5; refer Graphical abstract, C). Boron contents also changed in response to the nutritional treatment, with the lowest levels in the treatments with reduced quantities or low solubility Fe. Calcium levels increased in the treatment with 0.95 mM of Ca_3_(PO_4_)_2_ (LS-Pi), but this might be related to the calcium in the compound (Figure 5). Then, a partial effect of this ion in some of the observed results in the treatments with low solubility P cannot be discarded under this experimental design. The content of Fe was highly susceptible to the Fe in the media, with the plants treated with 25 μM HS-Fe or the LS-Fe source presenting 1% of the iron of the control plants (Figure 5; refer Graphical abstract, C). In most cases, the inoculation reduced the Fe content in plants, which might indicate a plant-bacteria competition for this resource (refer Graphical abstract, C). Additionally, as described in the literature (Hirsch et al., 2006; Bouain et al., 2019; Bechtaoui et al., 2021; Fan et al., 2021; Zheng and Liu, 2021), an interaction was observed between P and Fe, where the plants grown in P-deficiency presented up to 13% more of Fe than those in the P-control treatment (excepting the KT2440-inoculated plants that presented 47% less; Figure 5; refer Graphical abstract, C In turn, non-inoculated plants exposed to low-Fe presented a 13% less P than the control plants (refer Graphical abstract, C). Also, compared to the control plants, the N levels were lower in the non-inoculated plants treated with Fe deficiency (Figure 5). The PsJN and Sp7 strains reverted this effect, as the PsJN strain does not fix N, this phenomenon should not be related bacterial N-fixation.

In general, soils have less than 10 μM of Pi (as orthophosphate) available to plants (Wang et al., 2017; Luan et al., 2017). Inorganic forms of phosphorus account for 35%–70% of total phosphorus in soil (Guignard et al., 2017). Rhizospheric bacteria are the main ones responsible for solubilizing dicalcium phosphate, tricalcium phosphate, hydroxyl apatite, or organic P (Oleńska et al., 2020). Then, plants have adapted to thrive in P-starving conditions developing highly regulated mechanisms to regulate P levels under different nutritional conditions. Compared to the bulk soil, a decrease of 12% of available phosphorus has been suggested in the rhizosphere, with a corresponding increase in phosphatase activities, which indicates a hotspot with a high demand of P and plant-microorganisms competition for this nutrient (Liu et al., 2022). In this regard, using a synthetic community, Finkel et al. (2019) found that a deficiency in Pi changed the rhizospheric microbial community with the enrichment of *Burkholderia*, which correlates with a decreased Pi shoot content. Contrasting results were obtained here, where PsJN inoculated plants increased or maintained the P content in plants exposed to low solubility P o P deficiency (Figure 5).

Castrillo et al. (2017) found that the Arabidopsis microbiota was altered in *phr1/phl1* and *phf1* mutants in experiments using both natural and synthetic microbial communities. Finkel et al. (2019) found that *Burkholderia* sequences were enriched in low Pi in these mutants and in the wild-type plants, suggesting that this shift in their recruitment to the root under low Pi is independent of PSR activation. In the present study, the PsJN effects in Arabidopsis changed in the double mutant *phl1/phr1* depending on the P source. The PsJN strain significantly increased the rosette and root areas only when P was delivered mainly as LS-Pi (Figure 6). This might indicate that the effects of PsJN in plants growing in the low solubility P source would be independent of the PSR pathway. On the other hand, its effects on plants growing in HS-Pi would be dependent on this pathway (Figure 6). Interestingly, different genes related to PSR or P transport were differentially regulated by the inoculation with Sp7 or PsJN (i.e., *PHO1*, *PHT1;9*; Figure 7; refer Graphical abstract, B), and in some cases, the regulation was also affected by the P source (i.e., *miR399*; *PHF1-2*; Figure 7; refer Graphical abstract, B). An upregulation of *miR399* was previously reported by Sun et al. (2022) in *A. thaliana* plants under low P (using NaH_2_PO_4_ as a P source) and inoculated with *A. brasilense*.

As mentioned above, a connection between phytohormones and the mineral nutrient status in plants has been reported (e.g., Kuiper et al., 1989; Lamont et al., 2015). Then, the effects of inoculation and P source were analyzed in different genes related to auxin, ethylene, and JA. In the case of auxin, some genes were affected by the inoculation in an interaction with the P source, which might indicate a role for auxin in the differential observed phenotypical responses (i.e., *PILS3* and *DRM2/ARP*; Figure 8; refer Graphical abstract, B). In the case of JA and ethylene, the tested genes presented an upregulation induced by the inoculation, either with PsJN or Sp7, indicating that these hormonal pathways might be involved in general responses to these strains that do not depend on the P source (Supplementary Figure 2).

Many of the genes involved in Fe acquisition strategies are transcriptionally upregulated in response to Fe deficiency. In this scenario, Arabidopsis uses a reduction strategy where protons are released into the rhizosphere, resulting in local rhizosphere acidification (Satbhai et al., 2017). After acidification, Fe^3+^ is reduced to Fe^2+^ by FRO2 (FERRIC REDUCTASE OXIDASE 2). Then, Fe^2+^ is taken up into root epidermal cell layers by the specialized transporter IRT1 (IRON-REGULATED TRANSPORTER 1), which is essential for plant iron uptake from the soil (Vert et al., 2002). This process is regulated by FIT1 (ARABIDOPSIS FE-DEFICIENCY INDUCED TRANSCRIPTION FACTOR), which regulates FRO2 and IRT at the transcription level (Riaz and Guerinot, 2021). The results here confirm this regulation pattern. *FIT1* (*ARABIDOPSIS FE-DEFICIENCY INDUCED TRANSCRIPTION FACTOR*) was downregulated by Sp7 at 24 h in the highly soluble Fe form, coincidently with the downregulation of *FRO2.2* and *IRT1* at the same time and treatment (Figure 9). Interestingly, *IRT1* (*IRON-REGULATED TRANSPORTER 1*) was downregulated by both bacteria at 7d, irrespective of the iron treatment which might explain the lower Fe content that was detected in the inoculated plants in most of the treatments.

Carvalhais et al. (2013) used root exudates collected from maize plants grown under nitrogen (N), phosphate (P), iron (Fe), and potassium (K) deficiencies on the transcriptome of the PGPB *Bacillus amyloliquefaciens* FZB42. The largest shifts in gene expression patterns were observed in cells exposed to exudates from N-, followed by P-deficient plants. Exudates from P-deficient plants induced bacterial genes involved in chemotaxis and motility. This suggests that complex responses are induced in both partners in a plant-PGPB interaction in response to the nutritional context. The results presented here indicate that these complex responses are not only influenced by the nutrient concentrations but also by their availabilities, the elapsed time of the interaction, and the specific identities of the beneficial bacteria.

Altogether, the results presented here indicate that the plant responses to these proteobacteria are not only influenced by the nutrient (P and Fe) concentrations but also by their availabilities, the elapsed time of the interaction, and the specific identities of the beneficial bacteria. This provides valuable information to evaluate the interaction of plants with other beneficial strains and for applying beneficial bacteria in more complex contexts, such as in the field.

## Data availability statement

The raw data supporting the conclusions of this article will be made available by the authors, without undue reservation.

## Author contributions

MJP and DM conceived the experimental strategies. DM and DO performed the experiments shown throughout the manuscript, collected the data, and contributed to the experiment design and analysis of the results. MAI and JME characterized the mutant genotypes. MJP performed general interpretations of the results, analyzed the data, generated the graphics, and wrote the manuscript. DO, DM, MAI, JME, and MJP contributed to data interpretation and discussion and edited the manuscript. All authors contributed to the article and approved the submitted version.

## Funding

This work was funded by ANID PIA/BASAL FB0002, ANID-Millennium Science Initiative Program – NCN2021_010, ANID PIA/ANILLOS ACT210052, and ANID-FONDECYT 1190634. In addition, MAI was supported by ANID FONDECYT POSTDOCTORADO (grant 3220138). JME was also supported by ANPCyT (PICT2019-0015) and Fondo Nacional de Desarrollo Científico y Tecnológico (1200010).

## Conflict of interest

The authors declare that the research was conducted in the absence of any commercial or financial relationships that could be construed as a potential conflict of interest.

## Publisher’s note

All claims expressed in this article are solely those of the authors and do not necessarily represent those of their affiliated organizations, or those of the publisher, the editors and the reviewers. Any product that may be evaluated in this article, or claim that may be made by its manufacturer, is not guaranteed or endorsed by the publisher.

## Supplementary material

SUPPLEMENTARY TABLE 1 List and sequences of used primers.

SUPPLEMENTARY FIGURE 1 Effects of beneficial bacteria on phosphate homeostasis-related genes in *Arabidopsis thaliana* grown in different phosphate sources. Quantitative RT-PCR determinations of relative expression levels of the genes related to phosphate homeostasis *PHR1-1* (*PHOSPHATE STARVATION RESPONSE 1*); *PHL* (*PHR1-LIKE 1*); *PHT1;1* (*PHOSPHATE TRANSPORTER 1;1*); *PHT1;4*. (*PHOSPHATE TRANSPORTER 1;4*), and *PHO2* (*PHOSPHATE 2*). Plants were exposed to 1mm KH_2_PO_4_ and 0mM Ca_3_PO_4_ (Left panel) or 0.1mm KH_2_PO_4_/0.99mM Ca_3_PO_4_ (right panel) for 0, 2, 24 h or 7 days (d). Simultaneously, plants were non-inoculated (WB) or inoculated with *Paraburkholderia phytofirmans* PsJN (PsJN) or *Azospirillum brasilense* Sp7 (Sp7). Normalization was performed with the housekeeping *SAND* family gene (AT2G28390). Asterisks indicate statistical significance among treatments in a particular time compared to the WB group (Two-way ANOVA and multiple comparisons, p < 0.05).

SUPPLEMENTARY FIGURE 2 Effects of beneficial bacteria on jasmonic acid and ethylene-related genes in *Arabidopsis thaliana* grown in different phosphate sources. Quantitative RT-PCR determinations of relative expression levels of the genes related to auxin synthesis *LOX2* (*LIPOXYGENASE 2*) and *PDF1.2* (*PLANT DEFENSIN 1.2*. Plants were exposed to 1mm KH_2_PO_4_ and 0mM Ca_3_PO_4_; Left panel) or 0.1mm KH_2_PO_4_/0.99mM Ca_3_PO_4_ (right panel) for 0, 2, 24 h or 7 days (d). Simultaneously, plants were non-inoculated (WB) or inoculated with *Paraburkholderia phytofirmans* PsJN (PsJN) or *Azospirillum brasilense* Sp7 (Sp7). Normalization was performed with the housekeeping *SAND* family gene (AT2G28390). Asterisks indicate statistical significance among treatments in a particular time compared to the WB group (Two-way ANOVA and multiple comparisons, p < 0.05).

## References

[ref1] Abel S. Ticconi C. A. Delatorre C. A. (2002). Phosphate sensing in higher plants. Physiol. Plant. 115, 1–8. doi: 10.1034/j.1399-3054.2002.1150101.x 12010462

[ref2] Alzate Zuluaga M. Y. Martinez de Oliveira A. L. Valentinuzzi F. Tiziani R. Pii Y. Mimmo T.. (2021). Can inoculation with the bacterial biostimulant *Enterobacter* sp. strain 15S be an approach for the smarter P fertilization of maize and cucumber plants? Front. Plant Sci. 12:719873. doi: 10.3389/fpls.2021.719873, PMID: 34504509 PMC8421861

[ref3] An R. Moe L. A. (2016). Regulation of pyrroloquinoline quinone-dependent glucose dehydrogenase activity in the model rhizosphere-dwelling bacterium pseudomonas putida KT2440. Appl. Environ. Microbiol. 82, 4955–4964. doi: 10.1128/AEM.00813-16, PMID: 27287323 PMC4968544

[ref4] Barbez E. Kubes M. Rolcik J. Beziat C. Pencik A. Wang B.. (2012). A novel putative auxin carrier family regulates intracellular auxin homeostasis in plants. Nature 485, 119–122. doi: 10.1038/nature11001, PMID: 22504182

[ref5] Bari R. Datt Pant B. Stitt M. Scheible W. R. (2006). PHO2, microRNA399, and PHR1 define a phosphate-signaling pathway in plants. Plant Physiol. 141, 988–999. doi: 10.1104/pp.106.079707, PMID: 16679424 PMC1489890

[ref6] Barragán-Rosillo A. C. Peralta-Alvarez C. A. Ojeda-Rivera J. O. Arzate-Mejía R. G. Recillas-Targa F. Herrera-Estrella L. (2021). Genome accessibility dynamics in response to phosphate limitation is controlled by the PHR1 family of transcription factors in Arabidopsis. PNAS 118:e2107558118. doi: 10.1073/pnas.2107558118, PMID: 34385324 PMC8379931

[ref7] Bator I. Wittgens A. Rosenau F. Tiso T. Blank L. M. (2020). Comparison of three xylose pathways in *pseudomonas putida* KT2440 for the synthesis of valuable products. Front. Bioeng. Biotechnol. 7:480. doi: 10.3389/fbioe.2019.00480, PMID: 32010683 PMC6978631

[ref8] Bechtaoui N. Rabiu M. K. Raklami A. Oufdou K. Hafidi M. Jemo M. (2021). Phosphate-dependent regulation of growth and stresses Management in Plants. Front. Plant Sci. 12:679916. doi: 10.3389/fpls.2021.679916, PMID: 34777404 PMC8581177

[ref9] Bhosale R. Giri J. Pandey B. K. Giehl R. F. H. Hartmann A. Traini R.. (2018). A mechanistic framework for auxin dependent Arabidopsis root hair elongation to low external phosphate. Nat. Commun. 9:1409. doi: 10.1038/s41467-018-03851-3, PMID: 29651114 PMC5897496

[ref10] Bouain N. Krouk G. Lacombe B. Rouached H. (2019). Getting to the root of plant mineral nutrition: combinatorial nutrient stresses reveal emergent properties. Trends Plant Sci. 24, 542–552. doi: 10.1016/j.tplants.2019.03.008, PMID: 31006547

[ref11] Briat J. F. Fobis-Loisy I. Grignon N. Lobreaux S. Pascal N. Savino G.. (1995). Cellular and molecular aspects of iron metabolism in plants. Biol. Cell 84, 69–81. doi: 10.1016/0248-4900(96)81320-7

[ref12] Brumbarova T. Matros A. Mock H. P. Bauer P. (2008). A proteomic study showing differential regulation of stress, redox regulation and peroxidase proteins by iron supply and the transcription factor FER. Plant J. 54, 321–334. doi: 10.1111/j.1365-313X.2008.03421.x, PMID: 18221364

[ref13] Bustin S. A. Benes V. Garson J. A. Hellemans J. Huggett J. Kubista M.. (2009). The MIQE guidelines: minimum information for publication of quantitative real-time PCR experiments. Clin. Chem. 55, 611–622. doi: 10.1373/clinchem.2008.112797 19246619

[ref14] Bustos R. Castrillo G. Linhares F. Puga M. I. Rubio V. Pérez-Pérez J.. (2010). A central regulatory system largely controls transcriptional activation and repression responses to phosphate starvation in Arabidopsis. PLoS Genet. 6:e1001102. doi: 10.1371/journal.pgen.1001102, PMID: 20838596 PMC2936532

[ref15] Cappellari L. Del R. Santoro M. V. Schmidt A. Gershenzon J. Banchio E. (2019). Induction of essential oil production in Mentha x piperita by plant growth promoting bacteria was correlated with an increase in jasmonate and salicylate levels and a higher density of glandular trichomes. Plant Physiol. Biochem. 141, 142–153. doi: 10.1016/j.plaphy.2019.05.030, PMID: 31163341

[ref16] Carvalhais L. C. Dennis P. G. Fan B. Fedoseyenko D. Kierul K. Becker A.. (2013). Linking plant nutritional status to plant-microbe interactions. PLoS One 8:e68555. doi: 10.1371/journal.pone.0068555, PMID: 23874669 PMC3713015

[ref17] Castrillo G. Pereira Lima Teixeira P. J. Paredes S. H. Law T. F. Lorenzo L. D. Feltcher M. E.. (2017). Root microbiota drive direct integration of phosphate stress and immunity. Nature 543, 513–518. doi: 10.1038/nature21417, PMID: 28297714 PMC5364063

[ref1800] Ceasar S. A. Maharajan T. Hillary V. E. Ajeesh Krishna T. P. (2022). Insights to improve the plant nutrient transport by CRISPR/Cas system. Biotechnol. Adv. 59:107963. doi: 10.1016/j.biotechadv.2022.107963 35452778

[ref18] Chaudhary P. Singh S. Chaudhary A. Sharma A. Kumar G. (2022). Overview of biofertilizers in crop production and stress management for sustainable agriculture. Front. Plant Sci. 13:930340. doi: 10.3389/fpls.2022.930340, PMID: 36082294 PMC9445558

[ref19] Chea L. Pfeiffer B. Schneider D. Daniel R. Pawelzik E. Naumann M. (2021). Morphological and metabolite responses of potatoes under various phosphorus levels and their amelioration by plant growth-promoting Rhizobacteria. Int. J. Mol. Sci. 22:5162. doi: 10.3390/ijms22105162 34068175 PMC8153024

[ref20] Colombo C. Palumbo G. He J. Z. Pinton R. Cesco S. (2014). Review on iron availability in soil: interaction of Fe minerals, plants, and microbes. J. Soil. Sediment. 14, 538–548. doi: 10.1007/s11368-013-0814-z

[ref21] Compant S. Clément C. Sessitsch A. (2010). Plant growth-promoting bacteria in the rhizo- and endosphere of plants: their role, colonization, mechanisms involved and prospects for utilization. Soil Biol. Biochem. 42, 669–678. doi: 10.1016/j.soilbio.2009.11.024

[ref22] Connorton J. M. Balk J. Rodríguez-Celma J. (2017). Iron homeostasis in plants - a brief overview. Metallomics 9, 813–823. doi: 10.1039/c7mt00136c, PMID: 28686269 PMC5708359

[ref23] Crombez H. Motte H. Beeckman T. (2019). Tackling plant phosphate starvation by the roots. Dev. Cell 48, 599–615. doi: 10.1016/j.devcel.2019.01.002, PMID: 30861374

[ref24] Cruz-Hernández M. A. Mendoza-Herrera A. Bocanegra-García V. Rivera G. (2022). Azospirillum spp. from plant growth-promoting bacteria to their use in bioremediation. Microorganisms 10:1057. doi: 10.3390/microorganisms10051057, PMID: 35630499 PMC9143718

[ref25] Czechowski T. Stitt M. Altmann T. Udvardi M. K. Scheible W. R. (2005). Genome-wide identification and testing of superior reference genes for transcript normalization in Arabidopsis. Plant Physiol. 139, 5–17. doi: 10.1104/pp.105.063743, PMID: 16166256 PMC1203353

[ref26] Darrah P. (1993). The rhizosphere and plant nutrition: a quantitative approach. Plant and Soil 155-156, 1–20. doi: 10.1007/BF00024980

[ref27] Devi R. Kaur T. Kour D. Yadav A. Yadav A. N. Suman A.. (2022). Minerals solubilizing and mobilizing microbiomes: a sustainable approach for managing minerals’ deficiency in agricultural soil. J. Appl. Microbiol. 133, 1245–1272. doi: 10.1111/jam.15627, PMID: 35588278

[ref28] Donoso R. Leiva-Novoa P. Zúñiga A. Timmermann T. Recabarren-Gajardo G. González B. (2017). Biochemical and genetic bases of indole-3-acetic acid (auxin phytohormone) degradation by the plant growth-promoting rhizobacterium Paraburkholderia phytofirmans PsJN. Appl. Environ. Microbiol. 83, e01991–e01916. doi: 10.1128/AEM.01991-16, PMID: 27795307 PMC5165117

[ref29] Dorn E. Hellwig M. Reineke W. Knackmus H. J. (1974). Isolation and characterization of a 3-chlorobenzoate degrading pseudomonad. Arch. Microbiol. 99, 61–70. doi: 10.1007/BF00696222, PMID: 4852581

[ref30] Esmaeel Q. Miotto L. Rondeau M. Leclère V. Clément C. Jacquard C.. (2018). Paraburkholderia phytofirmans PsJN-plants interaction: from perception to the induced mechanisms. Front. Microbiol. 9:2093. doi: 10.3389/fmicb.2018.02093, PMID: 30214441 PMC6125355

[ref31] Fan X. Zhou X. Chen H. Tang M. Xie X. (2021). Cross-talks between macro- and micronutrient uptake and signaling in plants. Front. Plant Sci. 12:663477. doi: 10.3389/fpls.2021.663477, PMID: 34721446 PMC8555580

[ref32] Finkel O. M. Salas-González I. Castrillo G. Spaepen S. Law T. F. Teixeira P. J. P. L.. (2019). The effects of soil phosphorus content on plant microbiota are driven by the plant phosphate starvation response. PLoS Biol. 17:e3000534. doi: 10.1371/journal.pbio.3000534, PMID: 31721759 PMC6876890

[ref33] Fukami J. Cerezini P. Hungria M. (2018). Azospirillum: benefits that go far beyond biological nitrogen fixation. AMB Express 8, 73–12. doi: 10.1186/s13568-018-0608-1, PMID: 29728787 PMC5935603

[ref34] González E. Solano R. Rubio V. Leyva A. Paz-Ares J. (2005). PHOSPHATE TRANSPORTER TRAFFIC FACILITATOR1 is a plant-specific SEC12-related protein that enables the endoplasmic reticulum exit of a high-affinity phosphate transporter in Arabidopsis. Plant Cell 17, 3500–3512. doi: 10.1105/tpc.105.036640, PMID: 16284308 PMC1315384

[ref35] Graham P. Vance C. (2000). Nitrogen fixation in perspective: an overview of research and extension needs. Field Crop Res 65, 93–106. doi: 10.1016/S0378-4290(99)00080-5

[ref36] Gransee A. Merbach W. (2000). Phosphorus dynamics in a long-term P fertilization trial on Luvic Phaeozem at Halle. J. Plant Nutr. Soil Sci. 163, 353–357. doi: 10.1002/1522-2624(200008)163:4<353::AID-JPLN353>3.0.CO;2-B

[ref37] Guerinot M. L. Yi Y. (1994). Iron: nutritious, noxious, and not readily available. Plant Physiol. 104, 815–820. doi: 10.1104/pp.104.3.815 12232127 PMC160677

[ref38] Guignard M. S. Leitch A. R. Acquisti C. Eizaguirre C. Elser J. J. Hessen D. O.. (2017). Impacts of nitrogen and phosphorus: from genomes to natural ecosystems and agriculture. Front. Ecol. Evol. 5:70. doi: 10.3389/fevo.2017.00070

[ref39] Hammond J. P. Bennett M. J. Bowen H. C. Broadley M. R. Eastwood D. C. May S. T.. (2003). Changes in gene expression in Arabidopsis shoots during phosphate starvation and the potential for developing smart plants. Plant Physiol. 132, 578–596. doi: 10.1104/pp.103.020941, PMID: 12805589 PMC166999

[ref40] Han Y. White P. J. Cheng L. (2022). Mechanisms for improving phosphorus utilization efficiency in plants. Ann. Bot. 129, 247–258. doi: 10.1093/aob/mcab145, PMID: 34864840 PMC8835619

[ref41] Hirsch J. Marin E. Floriani M. Chiarenza S. Richaud P. Nussaume L.. (2006). Phosphate deficiency promotes modification of iron distribution in Arabidopsis plants. Biochimie 88, 1767–1771. doi: 10.1016/j.biochi.2006.05.007, PMID: 16757083

[ref42] Hong L. Orikasa Y. Sakamoto H. Ohwada T. (2019). Plant tissue localization and morphological conversion of Azospirillum brasilense upon initial interaction with Allium cepa L. Microorganisms. 7:275. doi: 10.3390/microorganisms7090275, PMID: 31438655 PMC6780411

[ref43] Israr D. Mustafa G. Khan K. S. Shahzad M. Ahmad N. Masood S. (2016). Interactive effects of phosphorus and pseudomonas putida on chickpea (*Cicer arietinum* L.) growth, nutrient uptake, antioxidant enzymes and organic acids exudation. Plant Physiol. Biochem. 108, 304–312. doi: 10.1016/j.plaphy.2016.07.023, PMID: 27485620

[ref44] Joshi H. Dave R. Venugopalan V. P. (2014). Pumping iron to keep fit: modulation of siderophore secretion helps efficient aromatic utilization in *pseudomonas putida* KT2440. Microbiology 160, 1393–1400. doi: 10.1099/mic.0.079277-0, PMID: 24742959

[ref45] Kim S. A. Guerinot M. L. (2007). Mining iron: iron uptake and transport in plants. FEBS Lett. 581, 2273–2280. doi: 10.1016/j.febslet.2007.04.043, PMID: 17485078

[ref46] Kobayashi T. Nishizawa N. (2012). Iron uptake, translocation, and regulation in higher plants. Annu. Rev. Plant Biol. 63, 131–152. doi: 10.1146/annurev-arplant-042811-105522 22404471

[ref47] Korir H. Mungai N. W. Thuita M. Hamba Y. Masso C. (2017). Co-inoculation effect of rhizobia and plant growth promoting rhizobacteria on common bean growth in a low phosphorus soil. Front. Plant Sci. 8:141. doi: 10.3389/fpls.2017.00141 28224000 PMC5293795

[ref48] Kuiper D. Kuiper P. J. C. Lambers H. Schuit J. Staal M. (1989). Cytokinin concentration in relation to mineral nutrition and benzyladenine treatment in Plantago major ssp. pleiosperma. Physiol. Plant. 75, 511–517. doi: 10.1111/j.1399-3054.1989.tb05617.x

[ref49] Kuzyakov Y. Blagodatskaya E. (2015). Microbial hotspots and hot moments in soil: concept and review. Soil Biol. Biochem. 83, 184–199. doi: 10.1016/j.soilbio.2015.01.025

[ref50] Lamont B. B. Pérez-Fernández M. Rodríguez-Sánchez J. (2015). Soil bacteria hold the key to root cluster formation. New Phytol. 206, 1156–1162. doi: 10.1111/nph.13228, PMID: 25534068

[ref51] Lay-Pruitt K. S. Wang W. Prom-U-Thai C. Pandey A. Zheng L. Rouached H. (2022). A tale of two players: the role of phosphate in iron and zinc homeostatic interactions. Planta 256:23. doi: 10.1007/s00425-022-03922-2, PMID: 35767117

[ref1001] Ledger T. Rojas S. Timmermann T. Pinedo I. Poupin M. J. Garrido T.. (2016). Volatile-mediated effects predominate in *Paraburkholderia phytofirmans* growth promotion and salt stress tolerance of *Arabidopsis thaliana*. Front. Microbiol. 7:1838. doi: 10.3389/fmicb.2016.01838 27909432 PMC5112238

[ref52] Liang G. (2022). Iron uptake, signaling, and sensing in plants. Plant Commun. 3:100349. doi: 10.1016/j.xplc.2022.100349, PMID: 35706354 PMC9483112

[ref53] Liu S. He F. Kuzyakov Y. Xiao H. Hoang D. T. T. Pu S.. (2022). Nutrients in the rhizosphere: a meta-analysis of content, availability, and influencing factors. Sci. Total Environ. 826:153908. doi: 10.1016/j.scitotenv.2022.153908, PMID: 35183641

[ref54] López-Arredondo D. L. Leyva-González M. A. González-Morales S. I. López-Bucio J. Herrera-Estrella L. (2014). Phosphate nutrition: improving low-phosphate tolerance in crops. Annu. Rev. Plant Biol. 65, 95–123. doi: 10.1146/annurev-arplant-050213-035949, PMID: 24579991

[ref55] López-Ruiz B. A. Zluhan-Martínez E. Sánchez M. P. Álvarez-Buylla E. R. Garay-Arroyo A. (2020). Interplay between hormones and several abiotic stress conditions on *Arabidopsis thaliana* primary root development. Cells 9:2576. doi: 10.3390/cells9122576, PMID: 33271980 PMC7759812

[ref1002] Luan M. Tang R. J. Tang Y. Tian W. Hou C. Zhao F.. (2017). Transport and homeostasis of potassium and phosphate: limiting factors for sustainable crop production. J. Exp. Bot. 68, 3091–3105. doi: 10.1093/jxb/erw444 27965362

[ref56] Mano Y. Nemoto K. (2012). The pathway of auxin biosynthesis in plants. J. Exp. Bot. 63, 2853–2872. doi: 10.1093/jxb/ers091 22447967

[ref57] Marín O. González B. Poupin M. J. (2021). From microbial dynamics to functionality in the rhizosphere: a systematic review of the opportunities with synthetic microbial communities. Front. Plant Sci. 12:650609. doi: 10.3389/fpls.2021.650609, PMID: 34149752 PMC8210828

[ref58] Miotto-Vilanova L. Jacquard C. Courteaux B. Wortham L. Michel J. Clément C.. (2016). Burkholderia phytofirmans PsJN confers grapevine resistance against *Botrytis cinere*a via a direct antimicrobial effect combined with a better resource mobilization. Front. Plant Sci. 7:1236. doi: 10.3389/fpls.2016.01236 27602036 PMC4993772

[ref59] Morrissey J. Guerinot M. L. (2009). Iron uptake and transport in plants: the good, the bad, and the ionome. Chem. Rev. 109, 4553–4567. doi: 10.1021/cr900112r, PMID: 19754138 PMC2764373

[ref60] Naqqash T. Hameed S. Imran A. Hanif M. K. Majeed A. van Elsas J. D. (2016). Differential response of potato toward inoculation with taxonomically diverse plant growth promoting Rhizobacteria. Front. Plant Sci. 7:144. doi: 10.3389/fpls.2016.00144, PMID: 26925072 PMC4756182

[ref61] Narang R. A. Bruene A. Altmann T. (2000). Analysis of phosphate acquisition efficiency in different Arabidopsis accessions. Plant Physiol. 124, 1786–1799. doi: 10.1104/pp.124.4.1786 11115894 PMC59875

[ref62] Naveed M. Qureshi M. A. Zahir Z. A. Hussain M. B. Sessitsch A. Mitter B. (2015). L-tryptophan-dependent biosynthesis of indole-3-acetic acid (IAA) improves plant growth and colonization of maize by *Burkholderia phytofirmans* PsJN. Ann. Microbiol. 65, 1381–1389. doi: 10.1007/s13213-014-0976-y

[ref63] Oleńska E. Małek W. Wójcik M. Swiecicka I. Thijs S. Vangronsveld J. (2020). Beneficial features of plant growth-promoting rhizobacteria for improving plant growth and health in challenging conditions: a methodical review. Sci. Total Environ. 743:140682. doi: 10.1016/j.scitotenv.2020.140682, PMID: 32758827

[ref64] Pegler J. L. Oultram J. M. J. Grof C. P. L. Eamens A. L. (2020). Molecular manipulation of the miR399/PHO2 expression module alters the salt stress response of *Arabidopsis thaliana*. Plants 10:73. doi: 10.3390/plants10010073, PMID: 33396498 PMC7824465

[ref65] Pinedo I. Ledger T. Greve M. Poupin M. J. (2015). *Burkholderia phytofirmans* PsJN induces long-term metabolic and transcriptional changes involved in *Arabidopsis thaliana* salt tolerance. Front. Plant Sci. 6:466. doi: 10.3389/fpls.2015.00466, PMID: 26157451 PMC4477060

[ref66] Poirier Y. Bucher M. (2001). Phosphate transport and homeostasis in Arabidopsis. Arabidopsis Book 1, 1–35. doi: 10.1199/tab.0024 PMC3243343 22303200

[ref67] Poirier Y. Jaskolowski A. Clúa J. (2022). Phosphate acquisition and metabolism in plants. Curr. Biol. 32, R623–R629. doi: 10.1016/j.cub.2022.03.073 35728542

[ref68] Poupin M. J. Greve M. Carmona V. Pinedo I. (2016). A complex molecular interplay of auxin and ethylene signaling pathways is involved in Arabidopsis growth promotion by *Burkholderia phytofir*mans PsJN. Front. Plant Sci. 7:492. doi: 10.3389/fpls.2016.00492, PMID: 27148317 PMC4828629

[ref69] Poupin M. J. Timmermann T. Vega A. Zuñiga A. González B. (2013). (2013) effects of the plant growth-promoting bacterium *Burkholderia phytofirmans* PsJN throughout the life cycle of *Arabidopsis thaliana*. PLoS One 8:e69435. doi: 10.1371/journal.pone.0069435, PMID: 23869243 PMC3711820

[ref70] Raghothama K. G. (1999). Phosphate acquisition. Annu. Rev. Plant. Physiol. Plant. Mol. Biol. 50, 665–693. doi: 10.1146/annurev.arplant.50.1.665 15012223

[ref71] Raghothama K. G. Karthikeyan A. S. (2005). Phosphate acquisition K. G. Plant and Soil 274, 37–49. doi: 10.1007/s11104-004-2005-6

[ref72] Rengel Z. Marschner P. (2005). Nutrient availability and management in the rhizosphere: exploiting genotypic differences. New Phytol. 168, 305–312. doi: 10.1111/j.1469-8137.2005.01558.x, PMID: 16219070

[ref73] Riaz N. Guerinot M. L. (2021). All together now: regulation of the iron deficiency response. J. Exp. Bot. 72, 2045–2055. doi: 10.1093/jxb/erab003, PMID: 33449088 PMC7966950

[ref74] Roslan M. A. M. Zulkifli N. N. Sobri Z. M. Zuan A. T. K. Cheak S. C. Abdul Rahman N. A. (2020). Seed biopriming with P- and K-solubilizing *Enterobacter hormaechei* sp. improves the early vegetative growth and the P and K uptake of okra Abelmoschus esculentus seedling. PLoS One 15:e0232860. doi: 10.1371/journal.pone.0232860, PMID: 32645001 PMC7347142

[ref75] Satbhai S. B. Setzer C. Freynschlag F. Slovak R. Kerdaffrec E. Busch W. (2017). Natural allelic variation of FRO2 modulates Arabidopsis root growth under iron deficiency. Nat. Commun. 8:15603. doi: 10.1038/ncomms15603, PMID: 28537266 PMC5458102

[ref76] Schachtman D. P. Reid R. J. Ayling S. M. (1998). Phosphorus uptake by plants: from soil to cell. Plant Physiol. 116, 447–453. doi: 10.1104/pp.116.2.447, PMID: 9490752 PMC1539172

[ref77] Schütz L. Gattinger A. Meier M. Müller A. Boller T. Mäder P.. (2018). Improving crop yield and nutrient use efficiency via biofertilization—a global meta-analysis. Front. Plant Sci. 8:2204. doi: 10.3389/fpls.2017.02204, PMID: 29375594 PMC5770357

[ref78] Somers E. Ptacek D. Gysegom P. Srinivasan M. Vanderleyden J. (2005). *Azospirillum brasilense* produces the auxin-like phenylacetic acid by using the key enzyme for indole-3-acetic acid biosynthesis. Appl. Environ. Microbiol. 71, 1803–1810. doi: 10.1128/AEM.71.4.1803-1810.2005, PMID: 15812004 PMC1082559

[ref79] Spaepen S. Bossuyt S. Engelen K. Marchal K. Vanderleyden J. (2014). Phenotypical and molecular responses of *Arabidopsis thaliana* roots as a result of inoculation with the auxin-producing bacterium Azospirillum brasilense. New Phytol. 201, 850–861. doi: 10.1111/nph.12590, PMID: 24219779

[ref80] Sun N. Huang L. Zhao H. Zhang N. Lin X. Sun C. (2022). Beneficial bacterium *Azospirillum brasilense* induces morphological, physiological and molecular adaptation to phosphorus deficiency in Arabidopsis. Plant Cell Physiol. 63, 1273–1284. doi: 10.1093/pcp/pcac101, PMID: 35859341

[ref81] Timmermann T. Armijo G. Donoso R. Seguel A. Holuigue L. González B. (2017). *Paraburkholderia phytofirmans* PsJN protects *Arabidopsis thaliana* against a virulent strain of *pseudomonas syringae* through the activation of induced resistance. MPMI 30, 215–230. doi: 10.1094/MPMI-09-16-0192-R, PMID: 28118091

[ref82] Timmermann T. Poupin M. J. Vega A. Urrutia C. Ruz G. A. González B. (2019). Gene networks underlying the early regulation of *Paraburkholderia phytofirmans* PsJN induced systemic resistance in Arabidopsis. PLoS One 14, e0221358–e0221324. doi: 10.1371/journal.pone.0221358, PMID: 31437216 PMC6705864

[ref83] Tiziani R. Mimmo T. Valentinuzzi F. Pii Y. Celletti S. Cesco S. (2020). Root handling affects carboxylates exudation and phosphate uptake of White Lupin roots [published correction appears in front]. Front. Plant Sci. 11:584568. doi: 10.3389/fpls.2020.584568, PMID: 33117414 PMC7566432

[ref84] Ullrich-Eberius C. I. Novacky A. van Bel A. J. E. (1984). Phosphate uptake in *Lemna gibba* G1: energetics and kinetics. Planta 161, 46–52. doi: 10.1007/BF00951459, PMID: 24253554

[ref85] Vance C. P. Uhde-Stone C. Allan D. L. (2003). Phosphorus acquisition and use: critical adaptations by plants for securing a nonrenewable resource. New Phytol. 157, 423–447. doi: 10.1046/j.1469-8137.2003.00695.x 33873400

[ref86] Vélez-Bermúdez I. C. Schmidt W. (2022). How plants recalibrate cellular iron homeostasis. Plant Cell Physiol. 63, 154–162. doi: 10.1093/pcp/pcab166, PMID: 35048128

[ref87] Vert G. A. Briat F. Curie C. (2003). Dual regulation of the Arabidopsis high-affinity root iron uptake system by local and long-distance signals. Plant Physiol. 132, 796–804. doi: 10.1104/pp.102.016089, PMID: 12805609 PMC167019

[ref88] Vert G. Grotz N. Dédaldéchamp F. Gaymard F. Guerinot M. L. Briat J. F.. (2002). IRT1, an Arabidopsis transporter essential for iron uptake from the soil and for plant growth. Plant Cell 14, 1223–1233. doi: 10.1105/tpc.001388, PMID: 12084823 PMC150776

[ref89] Von Uexküll H. R. Mutert E. (1995). Global extent, development and economic impact of acid soils. Plant and Soil 171, 1–15. doi: 10.1007/BF00009558

[ref90] Wang D. Lv S. Jiang P. Li Y. (2017). Roles, regulation, and agricultural application of plant phosphate transporters. Front. Plant Sci. 8:817. doi: 10.3389/fpls.2017.00817, PMID: 28572810 PMC5435767

[ref91] Wang Y. Wang F. Lu H. Liu Y. Mao C. (2021). Phosphate uptake and transport in plants: An elaborate regulatory system. Plant Cell Physiol. 62, 564–572. doi: 10.1093/pcp/pcab011, PMID: 33508131

[ref92] Wheal M. Fowles T. Palmer L. (2011). A cost-effective acid digestion method using closed polypropylene tubes for inductively coupled plasma optical emission spectrometry (ICP-OES) analysis of plant essential elements. Anal. Methods 3, 2854–2863. doi: 10.1039/c1ay05430a

[ref93] Williamson L. C. Ribrioux S. P. C. P. Fitter A. H. Leyser H. M. O. (2001). Phosphate availability regulates root system architecture in Arabidopsis. Plant Physiol. 126, 875–882. doi: 10.1104/pp.126.2.875, PMID: 11402214 PMC111176

[ref94] Wissuwa M. Gamat G. Ismail A. M. (2005). Is root growth under phosphorus deficiency affected by source or sink limitations? J. Exp. Bot. 56, 1943–1950. doi: 10.1093/jxb/eri189 15911558

[ref95] Wu P. Ma L. Hou X. Wang M. Wu Y. Liu F.. (2003). Phosphate starvation triggers distinct alterations of genome expression in Arabidopsis roots and leaves. Plant Physiol. 132, 1260–1271. doi: 10.1104/pp.103.021022, PMID: 12857808 PMC167066

[ref96] Yi C. Wang X. Chen Q. Callahan D. L. Fournier-Level A. Whelan J.. (2021). Diverse phosphate and auxin transport loci distinguish phosphate tolerant from sensitive Arabidopsis accessions. Plant Physiol. 187, 2656–2673. doi: 10.1093/plphys/kiab441, PMID: 34636851 PMC8644285

[ref97] Zak D. Kronvang B. Carstensen M. V. Hoffmann C. C. Kjeldgaard A. Larsen S. E.. (2018). Nitrogen and phosphorus removal from agricultural runoff in integrated buffer zones. Environ. Sci. Technol. 52, 6508–6517. doi: 10.1021/acs.est.8b01036, PMID: 29733209

[ref98] Zheng L. Huang F. Narsai R. Wu J. Giraud E. He F.. (2009). Physiological and transcriptome analysis of iron and phosphorus interaction in rice seedlings. Plant Physiol. 151, 262–274. doi: 10.1104/pp.109.141051, PMID: 19605549 PMC2735995

[ref99] Zheng Z. Liu D. (2021). SIZ1 regulates phosphate deficiency-induced inhibition of primary root growth of Arabidopsis by modulating Fe accumulation and ROS production in its roots. Plant Signal. Behav. 16:1946921. doi: 10.1080/15592324.2021.1946921, PMID: 34251993 PMC8330995

[ref100] Zúñiga A. Poupin M. J. Donoso R. Ledger T. Guiliani N. Gutiérrez R. A.. (2013). Quorum sensing and indole-3-acetic acid degradation play a role in colonization and plant growth promotion of *Arabidopsis thaliana* by *Burkholderia phytofirmans PsJN*. MPMI 26, 546–553. doi: 10.1094/MPMI-10-12-0241-R, PMID: 23301615

