# Supplementary material for: Plant-growth promotion by proteobacterial strains depends on the availability of phosphorus and iron in Arabidopsis thaliana plants
Source: Front Microbiol. 2022 Dec 13;13:1083270. doi: 10.3389/fmicb.2022.1083270 (PMC9792790; doi:10.3389/fmicb.2022.1083270)
Supplement: SUPPLEMENTARY TABLE 1 — List and sequences of used primers. [file Table_1.DOCX]

**Supplementary table 1:**

| **Locus (Name)** | **Primers (5’-3’)** | **Tm (ºC)** | **Amplicon (bp)** | **Reference** |
| --- | --- | --- | --- | --- |
| SAND (AT2G28390) | F: AACTCTATGCAGCATTTGATCCACT  R: TGATTGCATATCTTTATCGCCATC | 59 | 61 | [1] |
| miR399 (AT2G34202) | F: AATACTCCTATGGCAGATCGCATTGG  R: TCCTTTGGCAGAGAAGCATTTTACTTG | 59 | 62 | [2] |
| PHF1  (AT3G52190) | F: CAAGGAAGACTGCATCGACA  R: ATCTCCATCTTTCCCACCCA | 60 | 74 | Designed here |
| PHO1 (AT3G23430) | F: TAAGGAGATGGTGGGACGAA  R: TTAACCGTCTGAGTCCCTGTC | 58 | 503 | [3] |
| PHT1;9 (AT1G76430) | F: TGGAGCTGCAGGGAAGTTTG  R: ATCTGGAAAACCGTCCTCTTCAT | 58 | 88 | [4] |
| ASA1 (AT5G05730) | F: GAGCCAACGAGGCGTGGACC  R: GGCTCGAGCAAGACCAGCGG | 58 | 258 | [5] |
| PILS3 (AT1G76520) | F: GGAAGCTCCTCTCCGGGTGCT R: TAACGCGCAACCAAGACGCC | 61 | 156 | [5] |
| DRM2/ARP (AT2G33830) | F: GATCGGCGCGTAAGGAAAAC R: CCATTCCTCTAGTGGCGATGTTA | 58 | 72 | [5] |
| PHR1 (AT4G28610) | F: ACCTCAGATTGTTCAGCAGC  R: CAACAAAAGCCTCGTGAAGC | 60 | 134 | Designed here |
| PHL1 (AT5G29000) | F: TTCATCAGAGGACCAGCTCA  R: AATGCCTCGTGAAGTTCTGG | 60 | 96 | Designed here |
| PHT1;1 (AT5G43350) | F: TGATGATCTTGTGCTCTGTCG  R: ATGACACCCTTGGCTTCGT | 60 | 61 | [2] |
| PHT1;4 (AT2G38940) | F: CGAAGCTCCTCGGTCGTAT  R: GGAGAGTCCCAGGCTTTTGT | 58 | 60 | [2] |
| PHO2 (AT2G337709 | F: ACCGTTTCTCATCAAGGCGT  R: GTGCCCGTCCACCATAAGAA | 63 | 124 | [4] |
| LOX2 (At3g45140) | F: ATCAACGCTCGTGCACGCCA  R: CCGCGGGTAAGCCTTCCTGG | 64 | 142 | [6] |
| PDF1.2 (At5g44420) | F: CTTGTTCTCTTTGCTGCTTTCGAC  R: ATGCATTACTGTTTCCGCAAACC | 61 | 106 | [6] |
| FRO2 (AT1G01580) | F: CGCCACAAGAATCGCTCG  R: CTCCCACACTCGAACCTTCC | 59 | 99 | Designed here |
| IRT1 (AT4G19690) | F: CGAGCCTATACACCAGCAAG  R: CAAGAGCTGTGCATTTGACG | 59 | 125 | Designed here |
| FIT1 (AT2G28160) | F: GCCCCTGTTTCATAGACGAG  R: CGTTGGAGTCTAAGTCAGGC | 58 | 84 | Designed here |

**REFERENCES**

1. Czechowski, T., Stitt, M., Altmann, T., Udvardi, M. K., and Scheible, W. R. 2005. Genome-wide identification and testing of superior reference genes for transcript normalization in Arabidopsis. Plant Physiol. 139:5- 17.
2. Sun N, Huang L, Zhao H, Zhang N, Lin X, Sun C. 2022. Beneficial Bacterium Azospirillum brasilense Induces Morphological, Physiological and Molecular Adaptation to Phosphorus Deficiency in Arabidopsis. Plant Cell Physiol. 63(9):1273-1284. doi: 10.1093/pcp/pcac101. PMID: 35859341.
3. Stefanovic, A., Ribot, C., Rouached, H., Wang, Y., Chong, J., Belbahri, L., Delessert, S. and Poirier, Y. 2007. Members of the PHO1 gene family show limited functional redundancy in phosphate transfer to the shoot, and are regulated by phosphate deficiency via distinct pathways. The Plant Journal, 50: 982-994.
4. Pegler J.L, Oultram J.M.J, Grof C.P.L, Eamens A.L. 2021. Molecular Manipulation of the miR399/PHO2 Expression Module Alters the Salt Stress Response of Arabidopsis thaliana. Plants. 10(1):73.
5. Poupin M.J., Greve M, Carmona V and Pinedo I. 2016. A Complex Molecular Interplay of Auxin and Ethylene Signaling Pathways Is Involved in Arabidopsis Growth Promotion by Burkholderia phytofirmans PsJN. Front. in Plant Sci. 7:492.
6. Poupin, M.J., Timmermann, T., Vega, A., Zuniga, A., and Gonzalez, B. 2013. Effects of the plant growth-promoting bacterium Burkholderia phytofirmans PsJN throughout the life cycle of Arabidopsis thaliana. *PLoS One* 8, e69435.
