# Supplementary material for: Plant-growth promotion by proteobacterial strains depends on the availability of phosphorus and iron in Arabidopsis thaliana plants
Source: Front Microbiol. 2022 Dec 13;13:1083270. doi: 10.3389/fmicb.2022.1083270 (PMC9792790; doi:10.3389/fmicb.2022.1083270)
Supplement: SUPPLEMENTARY FIGURE 1 — Effects of beneficial bacteria on phosphate homeostasis-related genes in Arabidopsis thaliana grown in different phosphate sources. Quantitative RT-PCR determinations of relative expression levels of the genes related to phosphate homeostasis PHR1-1 (PHOSPHATE STARVATION RESPONSE 1); PHL (PHR1-LIKE 1); PHT1;1 (PHOSPHATE TRANSPORTER 1;1); PHT1;4. (PHOSPHATE TRANSPORTER 1;4), and PHO2 (PHOSPHATE 2). Plants were exposed to 1mm KH2PO4 and 0mM Ca3PO4 (Left panel) or 0.1mm KH2PO4/0.99mM Ca3PO4 (right panel) for 0, 2, 24 h or 7 days (d). Simultaneously, plants were non-inoculated (WB) or inoculated with Paraburkholderia phytofirmans PsJN (PsJN) or Azospirillum brasilense Sp7 (Sp7). Normalization was performed with the housekeeping SAND family gene (AT2G28390). Asterisks indicate statistical significance among treatments in a particular time compared to the WB group (Two-way ANOVA and multiple comparisons, p < 0.05). [file Data_Sheet_1.PDF]

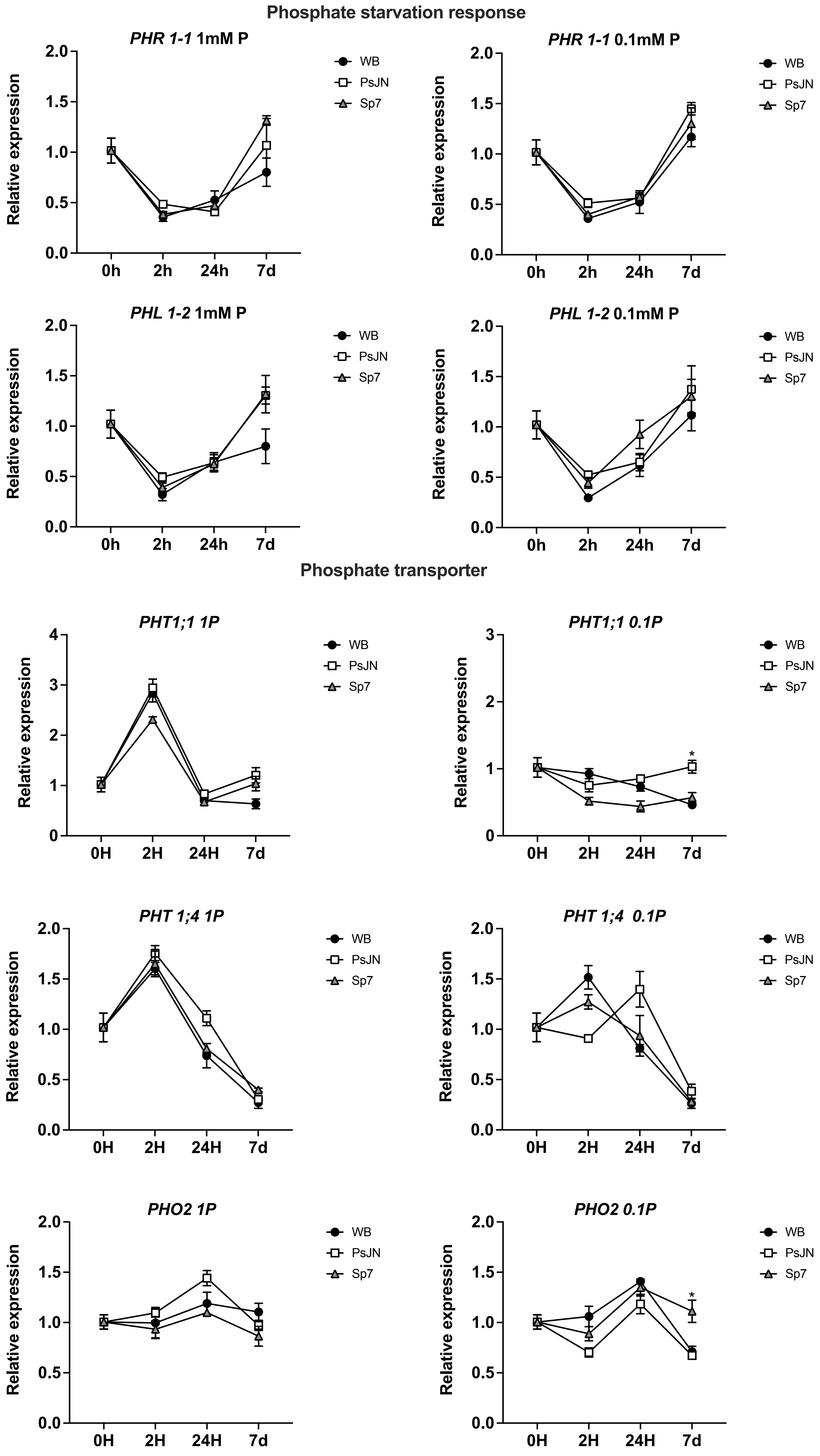

Phosphate starvation response
PHR 1-1 1mM P
PHR 1-1 0.1mM P
PHL 1-2 1mM P
PHL 1-2 0.1mM P
Relative expression
0.0
0.5
1.0
1.5
2.0
0h
2h
24h
7d
WB
PsJN
Sp7
Phosphate transporter
PHT1;1 1P
PHT1;1 0.1P
PHT 1;4 1P
PHT 1;4 0.1P
PHO2 1P
PHO2 0.1P
0
1
2
3
4
0H
2H
24H
7d
*
