# Supplementary figures and images for: Plant-growth promotion by proteobacterial strains depends on the availability of phosphorus and iron in Arabidopsis thaliana plants
Source: Front Microbiol. 2022 Dec 13;13:1083270. doi: 10.3389/fmicb.2022.1083270 (PMC9792790; doi:10.3389/fmicb.2022.1083270)

## Jasmonic acid synthesis

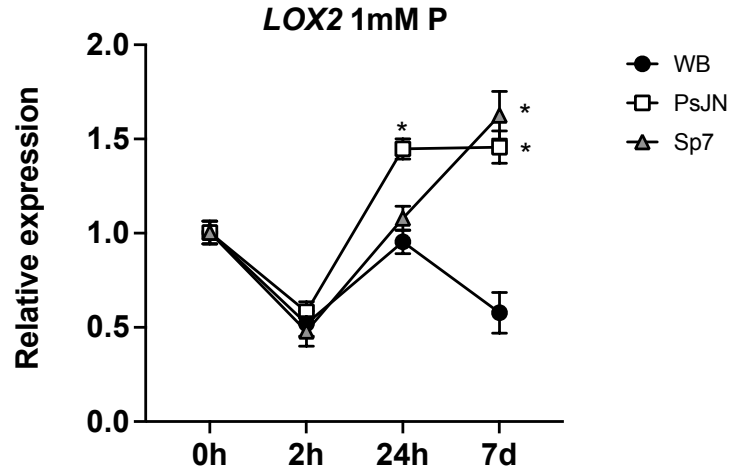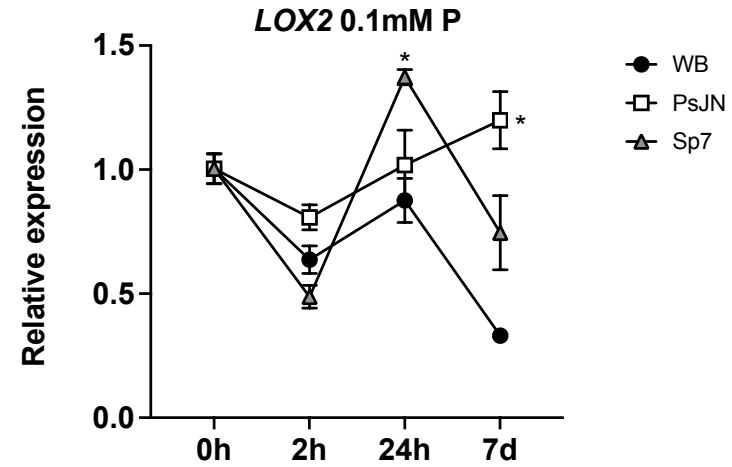

## Plant defense (Jasmonic acid and Ethylene)

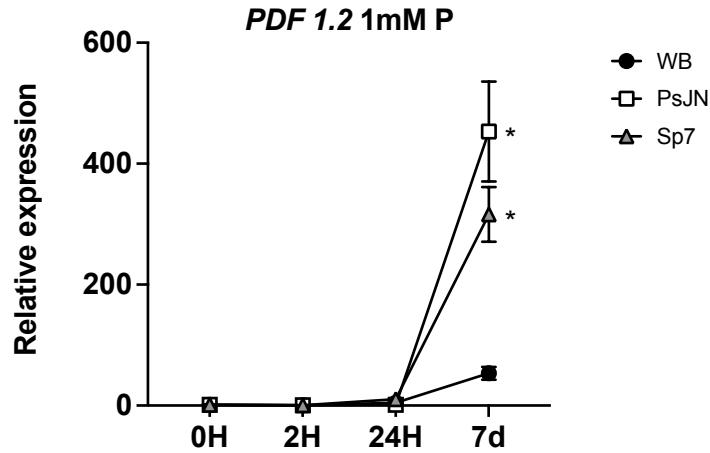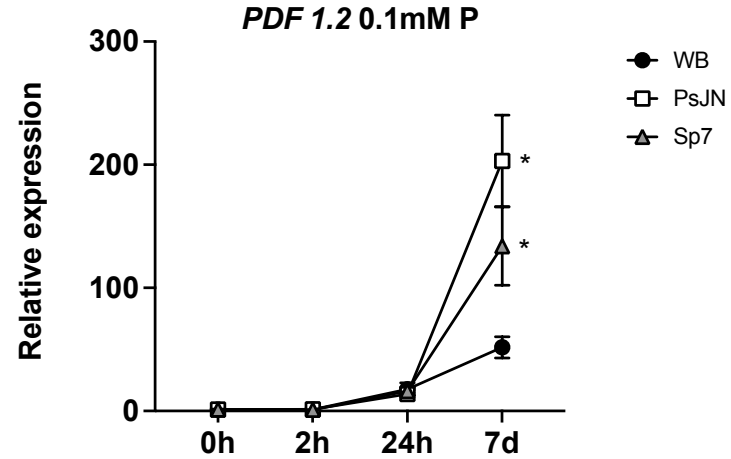

Supplement: SUPPLEMENTARY FIGURE 2 — Effects of beneficial bacteria on jasmonic acid and ethylene-related genes in Arabidopsis thaliana grown in different phosphate sources. Quantitative RT-PCR determinations of relative expression levels of the genes related to auxin synthesis LOX2 (LIPOXYGENASE 2) and PDF1.2 (PLANT DEFENSIN 1.2. Plants were exposed to 1mm KH2PO4 and 0mM Ca3PO4; Left panel) or 0.1mm KH2PO4/0.99mM Ca3PO4 (right panel) for 0, 2, 24 h or 7 days (d). Simultaneously, plants were non-inoculated (WB) or inoculated with Paraburkholderia phytofirmans PsJN (PsJN) or Azospirillum brasilense Sp7 (Sp7). Normalization was performed with the housekeeping SAND family gene (AT2G28390). Asterisks indicate statistical significance among treatments in a particular time compared to the WB group (Two-way ANOVA and multiple comparisons, p < 0.05). [file Data_Sheet_2.PDF]
